# Metabolic resource overlap impacts competition among phyllosphere bacteria

**DOI:** 10.1038/s41396-023-01459-0

**Published:** 2023-06-24

**Authors:** Rudolf O. Schlechter, Evan J. Kear, Michał Bernach, Daniela M. Remus, Mitja N. P. Remus-Emsermann

**Affiliations:** 1 Institute of Microbiology and Dahlem Centre of Plant Sciences, Department of Biology, Chemistry, Pharmacy, Freie Universität Berlin, Berlin, Germany; 2 School of Biological Sciences, University of Canterbury, Christchurch, 8011 New Zealand; 3 Biomolecular Interaction Centre, University of Canterbury, Christchurch, 8011 New Zealand; 4 Bioprotection Research Core, University of Canterbury, Christchurch, 8011 New Zealand; 5 Department of Electrical and Computer Engineering, University of Canterbury, Christchurch, 8011 New Zealand; 6 Protein Science and Engineering, Callaghan Innovation, School of Biological Sciences, University of Canterbury, Christchurch, New Zealand

**Keywords:** Microbial ecology, Microbial ecology, Comparative genomics

## Abstract

The phyllosphere is densely colonised by microbial communities, despite sparse and heterogeneously distributed resources. The limitation of resources is expected to drive bacterial competition resulting in exclusion or coexistence based on fitness differences and resource overlap between individual colonisers. We studied the impact of resource competition by determining the effects of different bacterial colonisers on the growth of the model epiphyte *Pantoea eucalypti* 299R (Pe299R). Resource overlap was predicted based on genome-scale metabolic modelling. By combining results of metabolic modelling and pairwise competitions in the *Arabidopsis thaliana* phyllosphere and in vitro, we found that ten resources sufficed to explain fitness of Pe299R. An effect of both resource overlap and phylogenetic relationships was found on competition outcomes in vitro as well as in the phyllosphere. However, effects of resource competition were much weaker in the phyllosphere when compared to in vitro experiments. When investigating growth dynamics and reproductive success at the single-cell resolution, resource overlap and phylogenetic relationships are only weakly correlated with epiphytic Pe299R reproductive success, indicating that the leaf’s spatial heterogeneity mitigates resource competition. Although the correlation is weak, the presence of competitors led to the development of Pe299R subpopulations that experienced different life histories and cell divisions. In some *in planta* competitions, Pe299R benefitted from the presence of epiphytes despite high resource overlap to the competitor strain suggesting other factors having stronger effects than resource competition. This study provides fundamental insights into how bacterial communities are shaped in heterogeneous environments and a framework to predict competition outcomes.

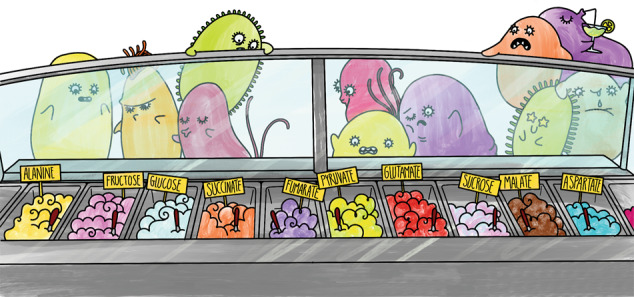

## Introduction

For bacteria, the leaf surface or the phyllosphere, is a challenging environment where resources are limited and heterogeneously distributed [1, 2]. However, leaves support bacterial populations of up to 10^7^ CFU per gram of leaf [3]. The number of CFU is impacted by the ability of bacteria to successfully colonise the heterogeneously distributed microenvironments on leaves. These microenvironments thereby influence local interactions and spatial structuring of bacterial communities [4–7].

Microbial communities exhibit intra- and inter-kingdom co-occurrence networks that are shaped and stabilised by priority effects and/or keystone microbial species [8, 9]. The interactions in these networks range from beneficial, neutral, to detrimental, resulting in increased, neutral, or decreased population densities, for at least one of the involved parties, compared to their monoculture [10]. Cooperative or beneficial microbial interactions include cross-feeding, biofilm formation, and cell communication; while competitive or detrimental interactions can be resource competition, contact-dependent antagonism, and secretion of toxic compounds [11]. Competition results in either exclusion or coexistence depending on the differences in the species’ niches and their fitness in an environment [12]. Competitive exclusion is driven by mechanisms that decrease niche differences and increase fitness differences between competing species. Resource use and preference are an important niche axis for a species, and the overlap or similarity with other species is a factor that balances coexistence and competitive exclusion through establishing a level of niche differentiation with others [13].

In the phyllosphere, a large number of bacterial interactions in a community context were shown to be negative [14]. The most parsimonious explanation for the negative interactions is the competition for resources and space, as secretion of antimicrobial compounds appears to be restricted to only a limited number of bacterial taxa in the phyllosphere [15]. Replacement series experiments revealed that pairs of near-isogenic epiphytic bacterial strains with high resource overlap exhibited a strong negative impact on each other’s population size on bean leaves [16]. By contrast, species pairs with a lower resource overlap resulted in larger population sizes than expected [16, 17]. However, this approach is not without its flaws, as it failed to predict competition outcomes between leaf-associated bacteria and a bacterial phytopathogen [18]. A challenge in defining a resource overlap is the lack of information of resource availability, use, and preference of a species in a specific environment. Genome-scale metabolic modelling allows for the study of the metabolic capabilities and nutrient requirements of members within microbial communities in defined growth conditions. Metabolic and community modelling has previously been used in an ecological context to understand the role of metabolic exchange in communities [19], the identifications of keystone species [20], and to define resource overlaps and cross-feeding potentials based on growth requirements [21–23].

As the phyllosphere is highly heterogeneous, “coarse-grained” investigations, such as those considering whole leaves or plants as the units of investigation, are not suited to study local interactions of leaf-associated bacteria. Therefore, to better understand bacterial growth dynamics in the phyllosphere, the micrometre or the single-cell scale must be the resolution of investigation, as every cell may experience a different fate such as microenvironments with different qualities and quantities of nutrients, or competitors [2, 3]. The intimate proximity of bacterial cells should thereby directly impact on community dynamics and short-distance interactions [3, 24, 25].

The CUSPER bioreporter (“repsuc” read backwards, from “reproductive success”), was developed in the epiphytic strain *Pantoea eucalypti* 299R (abb. Pe299R, syn. *Pantoea agglomerans* 299R, *Erwinia herbicola* 299R). Pe299R was originally isolated from a healthy Bartlett pear leaf and has been used in numerous studies since then to understand bacterial physiology and ecology in the phyllosphere [2, 5, 26–29]. Pe299R is part of the order Enterobacterales and the family *Erwiniaceae*. It is a copiotroph, utilises a wide range of nutrients, and grows optimally between 28 and 37 °C [17]. CUSPER reports on the number of divisions of individual cells from an initial population based on the dilution of a green fluorescent protein upon cell division and without de novo synthesis, and it has shown that Pe299R experience high variations of reproductive success in the phyllosphere [30, 31]. Due to the heterogeneously distributed and limited resources on leaves, the reproductive success of a bacterial cell depends on the local habitability. Consequently, it could be demonstrated that leaves that were pre-colonised with a near-isogenic Pe299R strain reduced the reproductive success of CUSPER bioreporter cells proportional to the pre-coloniser density [32]. However, interspecific competitions at the single-cell resolution in the phyllosphere have not been explored in such detail.

Here, we used genome-scale metabolic modelling to explain competition outcomes in defined growth conditions and in the phyllosphere for phylogenetically diverse leaf-associated bacterial isolates. Resource overlap was determined by metabolic modelling and expected to increase competition, leading to negative impacts on bacterial growth in vitro and *in planta*. To that end, the epiphyte *Pantoea eucalypti* 299R (Pe299R) was used as focal strain and challenged with six different phyllosphere-associated bacteria in pairwise competition experiments in different environmental contexts and scales.

## Materials and methods

### Bacterial strains and growth conditions

*Pantoea eucalypti* 299R (Pe299R) and representative epiphytic bacterial strains used in this study are listed in Table 1. Pe299R was used to construct the constitutively red fluorescent protein (mScarlet-I)-producing strain Pe299R::mSc, and was also the parental strain of the CUSPER bioreporter strain Pe299R_CUSPER_ (Pe299R::mSc (pProbe_CUSPER)), which harbours an additional IPTG-inducible green-fluorescent protein gene (Supplemental Materials and Methods). Bacteria were routinely grown on Reasoner’s 2a agar or broth (R2A, HiMedia, India) at 30 °C. Minimal media (MM) was used to evaluate growth and competition for defined carbon sources. Minimal media was composed of 1.62 g L^−1^ NH_4_Cl, 0.2 g L^−1^ MgSO_4_, 1.59 g L^−1^ K_2_HPO_4_, 1.8 g L^−1^ NaH_2_PO_4_·2H_2_O, with the following trace elements: 15 mg L^−1^ Na_2_EDTA·H_2_O, 4.5 mg L^−1^ ZnSO_4_·7H_2_O, 3 mg L^−1^ CoCl_2_·6H_2_O, 0.6 mg L^−1^ MnCl_2_, 1 mg L^−1^ H_3_BO_3_, 3.0 mg L^−1^ CaCl_2_, 0.4 mg L^−1^ Na_2_MoO_4_·2H_2_O, 3 mg L^−1^ FeSO_4_·7H_2_O, and 0.3 mg L^−1^ CuSO_4_·5H_2_O [33]. Carbon sources used were glucose, fructose, sorbitol, malate, and methanol for determining carbon utilisation profiles and in vitro competition assays.

**Table 1:** Phyllosphere-associated bacterial strains.

Phylum	Phylogroup	PD	Species	Abbr.
Pseudomonadota	Gammaproteobacteria	n.a.	*Pantoea eucalypti* 299R	Pe299R
		0.41	*Pseudomonas koreensis* P19E3	PkP19E3
			*Pseudomonas syringae* pv. *syringae* B728a	PssB728a
	Alphaproteobacteria	0.68	*Methylobacterium* sp. Leaf85	MethL85
			*Sphingomonas melonis* FR1	SmFR1
Actinomycetota	Actinobacteria	0.76	*Arthrobacter* sp. Leaf145	ArthL145
			*Rhodococcus* sp. Leaf225	RhodL225

Phylogenetic distances (PD) are in relation to Pe299R. *N.a.* Not applicable.

### Phylogeny

A phylogenetic tree for the seven phyllosphere-associated strains was constructed based on a multiple sequence alignment of a set of concatenated 31 single-copy genes [34]. Alignment was done with MAFFT, and a phylogenetic tree was then inferred using UPGMA with a Jukes-Cantor model. Concatenation, alignment, and tree inference were performed in Geneious Prime 2022.2.2 (https://www.geneious.com). Newick files were exported into R to retrieve a phylogenetic distance matrix based on branch lengths between strains and Pe299R, using the package *ape* [35].

### In vitro growth assays

Each strain was grown at 30 °C in R2A broth until the late stationary phase. Cells were then harvested by centrifugation at 2000 × *g* for 5 min, washed twice in phosphate buffer saline (PBS, 0.2 g L^−1^ NaCl, 1.44 g L^−1^ Na_2_HPO_4_ and 0.24 g L^−1^ KH_2_PO_4_), and resuspended in MM to an optical density at 600 nm (OD_600_) of 0.5. Afterwards, 20 µL of bacterial suspension were added to 180 µL of MM supplemented with a carbon source in flat bottom 96-well microtiter plates (Costar, Corning, NY, USA) with four technical replicates per condition. Carbon utilisation profiles for each species was determined by supplementing MM with a final concentration of 0.2% w/v of a sole carbon source (glucose, fructose, sorbitol, or malate), or 0.2% v/v of methanol. Minimal medium without added carbon source was used as a negative control. The microtiter plates were sealed with a breathable membrane (4ti-0516/96; gas permeability of 0.6 m^3^ m^−2^ day^−1^ and water loss of 1 g m^−2^day^−1^; Brooks Life Sciences, UK), and incubated at 30 °C with shaking. Optical density was measured in a FLUOstar Omega microplate reader (BMG Labtech Ltd., UK) for up to 5 days in 24 h intervals on the same batch culture. For each measurement, ten measurements in different positions of each well were recorded and averaged. The experiments were conducted twice independently. Growth curves of each strain in each growth condition were first normalised by the growth curve in minimal media without carbon source and then used to estimate growth rate (μ), carrying capacity (K), and area under the curve (AUC), by fitting logistic growth curves using the *R* package *growthcurver* [36]. The accuracy of the μ estimations were determined by fitting a logistic model to growth data of Pe299R in nutrient broth at different temporal resolution (Fig. S1). These values were grouped using complete-linkage hierarchical clustering based on the Euclidean distance matrix between species, in which the distance between Pe299R and a second species was used as a proxy of carbon utilisation dissimilarity.

### Construction of genome-scale metabolic and community models

Genomes were retrieved from the PATRIC database, and the annotation files were used to create either individual metabolic models or two-species communities, in which Pe299R was always present, in CarveMe [37]. Models were gap filled using a minimal media composition with (1) carbon sources used for carbon utilisation profiles, or (2) carbon sources detected in *Arabidopsis thaliana* leaves [38, 39]. An index of metabolic resource overlap (MRO) was calculated for each two-species community model using the “species metabolic interaction analysis” (SMETANA) modelling approach developed by Zelezniak et al. [22]. MRO was calculated based on a in silico media composition that emulates the growth media tested empirically (Table S1). Similar to the in vitro experiments, the composition of the in silico media included glucose, fructose, malate, sorbitol, and methanol (M5C). To determine the MRO between pair of species in the phyllosphere, five different media compositions were specified a priori based on a range of carbon sources identified in *Arabidopsis thaliana* leaves [38, 39], as the composition of the available resources in the phyllosphere is not yet well defined (L8C, L10C, L13C, L18C, and L26C), detailed in Table S1 [38, 39]. Methanol was included in every media composition, as it is detected on leaf surfaces and is a relevant source of carbon for phyllosphere-associated methylotrophs [40, 41]. Construction of metabolic models and MRO were performed using the High-Performance Computer at ZEDAT, Freie Universität Berlin [42].

### Competition for carbon sources

Competition assays were performed in MM supplemented with mixed carbon sources (MM_5×C_), composed of a total 0.125% w/v of glucose, fructose, sorbitol, malate, and methanol (0.025% w/v glucose, 0.025% w/v fructose, 0.025% w/v malate, 0.025% w/v sorbitol, and 0.025% v/v methanol). The red fluorescent Pe299R::mSc strain was competed against individual non-fluorescent bacteria by mixing both strains in a 1:1 OD_600_ ratio, as described previously [43]. The fitness of Pe299R::mSc is comparable to its non-fluorescent parental strain [43]. Briefly, flat bottom 96-well microtiter plates (Costar, Corning, NY, USA) were seeded with 200 µL MM_5×C_ containing a defined mixed bacterial suspension (final OD_600_ = 0.05, three technical replicates, one independent experiment). The microplate was sealed with a breathable membrane (4ti-0516/96; gas permeability of 0.6 m^3^m^−2^ day^−1^ and water loss of 1 g m^−2^ day^−1^; Brooks Life Sciences, UK), incubated at 30 °C with shaking in a microplate reader, and the red fluorescence of Pe299R::mSc was measured every 5 min for 20 h using an excitation filter at 584 nm and an emission filter at 620–10 nm. Growth parameters from fluorescence growth curves (μ_RFU_, K_RFU_, AUC_RFU_) were estimated in *growthcurver*. A competitive ability score based on Chesson’s framework of Coexistence Theory [44] was calculated as in Eq. 1.

1 $$Competitive\,score = \frac{{\mu _i - 1}}{{\sqrt {a_{ii}a_{ij}} }}$$

Where *μ*_*i*_ is the estimated growth rate of Pe299R::mSc in monoculture, *a*_*ii*_ is the competition coefficient of Pe299R::mSc when a near-isogenic Pe299R strain is present (intraspecific competition), and *a*_*ij*_ is the competition coefficient of Pe299R::mSc when a different strain is present (interspecific competition). Competition coefficients were calculated as the reciprocal of the corresponding K [45]. For simplicity, competitive ability scores were rescaled and centred to zero (z-score).

### Plant growth

Arabidopsis seeds (*Arabidopsis thaliana* Col-0) were surface-sterilised in a solution containing 50% v/v ethanol and 6% v/v H_2_O_2_ for 90 s, then thoroughly washed three times with sterile distilled water. Before sowing, seeds were stratified in sterile water at 4 °C in the dark for at least 2 days. Four seeds were sown aseptically in tissue-culture vessels (Magenta GA-7, Magenta LLC., IL, USA) containing 50 mL of ½ Murashige & Skoog (MS; Duchefa, The Netherlands) agar media (1.0% w/v, pH 5.9) in sterile conditions. For gas exchange, the lids of the Magenta GA-7 tissue-culture boxes featured four 1 cm diameter holes that were covered with two layers of 3 M Micropore tape [46]. Plants were grown in a Conviron A1000 plant growth chamber at 22 °C, 80% relative humidity and short-day photoperiod (11 h day cycles, light intensity ~120–150 µE m^−2^ s^−1^).

### Plant inoculation

For plant inoculation, an exponentially growing Pe299R_CUSPER_ culture in lysogeny broth (LB; HiMedia, India) supplemented with 50 µg mL^−1^ kanamycin was induced with 1 mM isopropyl beta-D-1-thiogalactopyranoside (IPTG), as described in detail in Supplemental Material and Methods. Competitor strains were grown on R2A agar plates for 2–5 days, depending on the strain, and a loop of bacteria was resuspended and washed twice in PBS. The IPTG-induced Pe299R_CUSPER_ and the bacterial suspensions were mixed in a 1:1 ratio and adjusted to a final OD_600_ of 0.005. Four-week-old arabidopsis plants were inoculated with 200 µL of the bacterial mix per box using a sterile airbrush (KKmoon Airbrush Model T130A). Plants were harvested at 0, 24, 36, and 48 h post-inoculation (hpi) by cutting the complete leaf rosette from the roots using sterile scissors and scalpel, and transferring the plant into a 1.7 mL microcentrifuge tube. Four individual plants were used per condition (one independent experiment). After the fresh weight of each plant was determined, 1 mL PBS with 0.02% v/v Silwet L-77 was added. Samples were shaken in a bead mill homogenizer (Omni Bead Ruptor 24, Omni International, GA, USA) for two cycles of 5 min at a speed of 2.6 m s^−1^, and sonicated for 5 min (Easy 30 H, Elmasonic, Elma Schmidbauer GmbH, Germany).

Leaf washes were plated onto R2A (total bacterial density) and R2A supplemented with 15 µg mL^−1^ gentamicin (Pe299R_CUSPER_ population), and CFU were determined by serial dilutions and normalised by the corresponding plant fresh weight (CFU gFW^−1^). Growth curve parameters from CFU of Pe299R (μ, K) were used to calculate the competitive ability of an epiphytic strain against Pe299R, as previously described (Eq. 1). The remaining supernatants were transferred into a sterile 1.7 mL microcentrifuge tube and centrifuged at 15,000 × *g* for 10 min at 4 °C to collect cells for microscopy. Cells were resuspended and fixed in 100 µL of fixative solution (4% w/v paraformaldehyde –PFA– in PBS) overnight at 4 °C. After this period, cells were washed twice in PBS and resuspended in 20 µL PBS. Then, one volume of 96% v/v ethanol was added to the samples. Samples were stored at −20 °C until further analysis.

### Microscopy

Cells recovered from leaves after 0, 24, and 36 hpi were mounted on microscopy slides coated with 0.1% w/v gelatine. Images were acquired on a AxioImager.M1 fluorescent widefield microscope (Zeiss) at 1000× magnification (EC Plan-Neofluar 100×/1.30 Ph3 Oil M27 objective) equipped with the Zeiss filter sets 38HE (BP 470/40-FT 495-BP 525/50) and 43HE (BP 550/25-FT 570-BP 605/70), an Axiocam 506 (Zeiss), and the software Zen 2.3 (Zeiss). At least 100 cells were acquired per biological replicate in three different channels: green (38HE filter set), red (43HE filter set), and phase contrast.

### Image analysis

Images were analysed in FIJI/ImageJ v. 2.0.0-rc-69/1.52 s [47]. As Pe299R_CUSPER_ constitutively expresses mScarlet-I, the red fluorescence was used to identify Pe299R_CUSPER_ cells, including daughter cells with low green fluorescence, using the thresholding method “intermodes” and converted into a binary mask object. Only particles in a size range of 0.5–2.5 µm were considered, excluding cells on the edges of the field of view. All objects were manually inspected using the phase contrast images to corroborate the selection of bacterial cells, and to exclude false positive red fluorescent particles. The mask was then used to determine green fluorescence of Pe299R_CUSPER_ cells in the green-fluorescent channel to calculate the reproductive success (RS) of single cells. In addition, background fluorescence was measured by sampling a random section of background area in each fluorescent image [2].

### Estimation of single-cell reproductive success

The RS of the Pe299R_CUSPER_ bioreporter is calculated as the number of divisions a GFP-loaded cell underwent after arrival to a new environment. This estimation is based on the dilution of the green-fluorescent signal after cell division, as de novo biosynthesis of GFP is transcriptionally repressed [30]. The RS of Pe299R_CUSPER_ cell at a time *t* was estimated from background-corrected fluorescence measurements by subtracting the mean background fluorescence from the mean fluorescence intensity of each cell in each field of view. Then, the reproductive success of a cell *n* at time *t* (RS_n,t_)—number of divisions of an immigrant cell since its arrival to a new environment—was calculated as

2 $$RS_{n,t} = log_2\left( {\frac{{\overline {{\it{x}}_0} }}{{{\it{x}}_{n,t}}}} \right)$$

Where $\overline {{\it{x}}_0}$ is the mean intensity of the cell population at time zero, and *x*_*n,t*_ the fluorescence intensity of a single cell *n* at time *t* [31].

As the Pe299R_CUSPER_ bioreporter decreases in intensity upon each cell division, the RS value from the background intensity measurements in the green-fluorescent channel was calculated to define a limit of detection (LOD) for Pe299R_CUSPER_. The LOD was defined as the RS value that has a 5% probability of being background noise. Consequently, calculated values of RS for single cells above this threshold were grouped, as the number of generations that a cell with low fluorescent intensity underwent cannot be further estimated. The distribution of RS from the initial cell population was determined as a relative fraction of Pe299R_CUSPER_ cells from the total observed population, by binning cells into subpopulations with different RS values: RS_0_: RS < 0.5; RS_1_: 0.5 ≤ RS < 1.5; RS_2_: 1.5 ≤ RS < 2.5; RS_3_: 2.5 ≤ RS < 3.5; RS_4_: 3.5 ≤ RS < 4.5; RS_>4_: RS ≥ 4.5. Non-Metric Multidimensional Scaling (NMDS) and Permutational Multivariate Analysis of Variance (PERMANOVA) with Bray-Curtis dissimilarities was selected to evaluate the variation of a Pe299R_CUSPER_ population (as relative fractions) explained by multivariate data (i.e., time of sampling and presence of an epiphyte). Bray-Curtis dissimilarity matrix and PERMANOVA with 999 permutations were performed using the *vegan* package [48].

### Data analysis

If not stated otherwise, all data processing, statistical analyses, and graphical representation were performed in R [49]. Data processing and visualisation were performed using the *tidyverse* package [50]. Graphical representations of matrices were constructed using the *ComplexHeatmap* package [51]. Pearson’s correlations (*r*) were used to compare variables using the *cor()* function of the *stats* package. Linear or generalised linear regressions were constructed with the *lm()* or *glm()* function from the *stats* package, respectively, to evaluate the effect of the presence of an epiphyte, metabolic resource overlap (MRO), and/or time of sampling with the competitive ability of a strain against Pe299R in different environments (in vitro and in the phyllosphere). ANOVA were performed using the *aov()* function of the *stats* package. Distribution of the residuals in each model were inspected visually and homoscedasticity was evaluated with the Breusch-Pagan test using the *ncvTest()* function of the *car* package. Eta squared (η^2^) was used to measure the effect size of the predictors in the regression models using the *lsr* package [52].

## Results

### Differences in carbon utilisation are predicted by genome-scale metabolic modelling

**Fig. 1:**
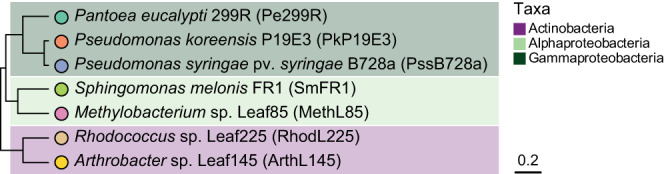
Phylogenetic tree of phyllosphere-associated bacterial strains. An UPGMA tree was created for the strains used in this study using a set of 31 single-copy marker genes [34]. Scale bar represents the number of substitutions per site.

Differences in growth on different carbon sources and the metabolic resource overlap (MRO) between the focal species *Pantoea eucalypti* 299R (Pe299R) and members of actinobacteria, gamma-, and alphaproteobacteria (Fig. 1) were determined empirically and based on genome-scale metabolic models, respectively.

**Fig. 2:**
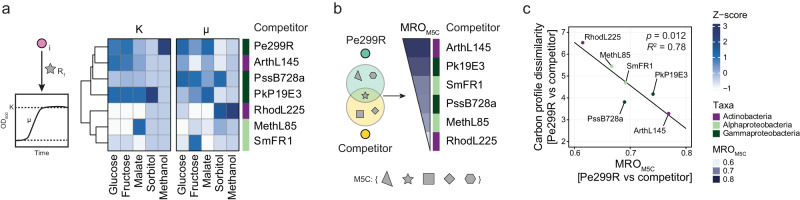
Metabolic resource overlap portrays differences in empirical carbon utilisation profiles between Pe299R and a second strain. **a** Carbon utilisation matrix. Bacterial strains were clustered based on estimated carrying capacity (K) and growth rates (μ) from growth in minimal medium supplemented with individual carbon sources selected for validation of in silico analysis. Values were rescaled into z-scores for complete-linkage clustering. **b** Metabolic resource overlap (MRO) is an index of resource similarity modelled, based on genomic information, under in silico media composition including glucose, fructose, malate, sorbitol, and methanol (M5C). Strains were ranked based on descending MRO_M5C_ with Pe299R. **c** Linear relationship of MRO_M5C_ and carbon profile dissimilarity between Pe299R and a second strain.

Individual strains were grown in MM supplemented with either 0.2% w/v glucose, fructose, malate, sorbitol, or 0.2% v/v methanol and OD_600_ was measured in 24 h intervals for five consecutive days (Fig. S2a). Estimated growth rate (μ) and carrying capacity (K) were retrieved for each growth curve and used to cluster the strains based on similarity (Fig. 2a, Table S2). Complete-linkage hierarchical clustering based on utilised resources placed Pe299R in a clade with the gammaproteobacteria *Pseudomonas syringae* pv. *syringae* B728a (PssB728a), *Pseudomonas koreensis* P19E3 (PkP19E3), and the actinobacterium *Arthrobacter* sp. Leaf145 (ArthL145). These strains were able to grow on glucose, fructose, and malate, reaching high and similar K in liquid media. PssB728a was also able to grow in sorbitol. The most dissimilar strains in relation to Pe299R were *Sphingomonas* *melonis* FR1 (SmFR1), *Rhodococcus* sp. Leaf225 (RhodL225), and *Methylobacterium* sp. Leaf85 (MethL85). The resulting carbon utilisation profile was used as an empirical distance matrix for resource use dissimilarity between Pe299R and a second strain.

The MRO is an estimation of the maximal overlap between the minimal growth requirements of two (or more) metabolic models [22]. Thus, MRO calculates the potential of species to compete for a list of compounds defined a priori. From a list that includes glucose, fructose, malate, sorbitol, and methanol as carbon sources (M5C), MRO_M5C_ was calculated for Pe299R and secondary strains (Fig. 2b, Table 2). The strain pair Pe299R–ArthL145, as well as Pe299R–PkP19E3 showed the highest MRO_M5C_ values (0.77 and 0.74, respectively), which were part of the same cluster based on empirical growth profile, while the lowest MRO_M5C_ and highest profile dissimilarity were observed between the pairs Pe299R–MethL85 and Pe299R–RhodL225 (MRO_M5C_ of 0.67 and 0.62, respectively). The carbon profile dissimilarity between Pe299R and a second strain was strongly correlated with MRO_M5C_ (*r* = −0.91, *p* = 0.012) but not with phylogenetic distances (*r* = 0.41, *p* = 0.42). Additionally, MRO_M5C_ was a predictor of carbon profile dissimilarity between the focal and other strains (Fig. 2c). Linear regression analysis showed a negative relationship between MRO and carbon profile dissimilarity (*R*^2^ = 0.78, *F*_1,4_ = 18.77, *p* = 0.012). This relationship was mainly due to the dissimilarity in estimated growth rates (Fig. S3, *R*^2^ = 0.80, *F*_1,2_ = 21.57, *p* = 0.0097). The presented results suggest that genome-scale metabolic modelling can be used to explain strain differences in resource use in a defined environment.

**Table 2:** Dissimilarity metrics and competition scores of phyllosphere-associated strains in relation to Pe299R.

Strain	in vitro			*in planta*					
	Carbon profile dissimilarity	MRO_M5C_	Competition score	MRO_L8C_	MRO_L10C_	MRO_L13C_	MRO_L18C_	MRO_L26C_	Competition score
PkP19E3	4.17	0.74	2.64	0.64	0.64	0.64	0.64	0.69	−0.75
PssB728a	3.81	0.69	3.03	0.59	0.67	0.59	0.59	0.77	1.31
MethL85	5.45	0.67	0.92	0.69	0.69	0.62	0.69	0.69	0.81
SmFR1	4.69	0.69	1.05	0.64	0.64	0.62	0.62	0.88	−1.03
ArthL145	3.28	0.77	2.41	0.69	0.81	0.85	0.81	0.81	0.51
RhodL225	6.53	0.62	0.69	0.69	0.62	0.69	0.69	0.77	−0.86

Carbon profile dissimilarities and MRO are in relation to Pe299R

### Competition in vitro is driven by resource overlaps

**Fig. 3:**
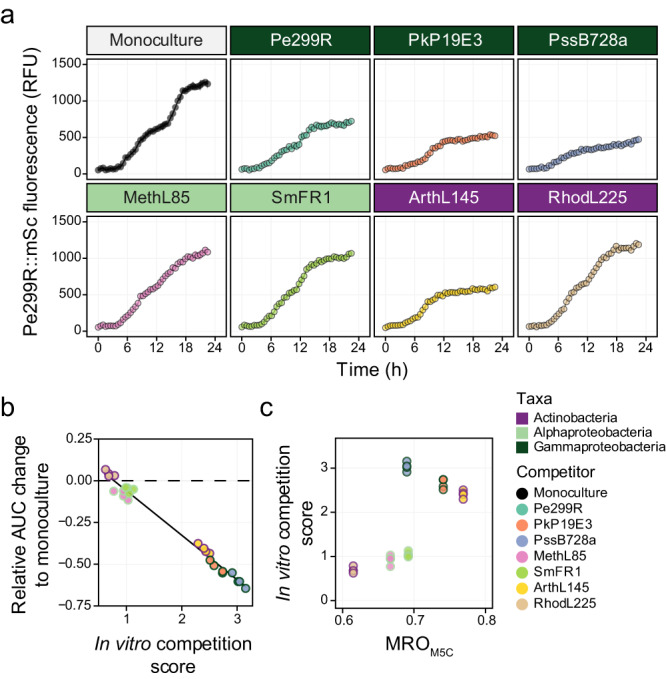
Pe299R is affected by the presence of a competitor in vitro. **a** Fluorescence curves of Pe299R::mSc co-inoculated with a competitor (top label) in MM_5×C_. **b** Relationship between competition score (Eq. 1) and the relative change in the area under the fluorescent curve of Pe299R::mSc in the presence of a competitor in relation to the monoculture. **c** Relationship between MRO_M5C_ and the competitive score of a second epiphyte against Pe299R. Details of the regression model can be found in Table S4.

To confirm the predictions of the genome-scale metabolic modelling, the ability of a competitor to affect the growth of a fluorescently red-labelled Pe299R strain (Pe299R::mSc) was evaluated in vitro. First, the optical density of every strain was measured in MM supplemented with multiple resources (MM_5×C_: 0.025% w/v glucose, 0.025% w/v fructose, 0.025% w/v malate, 0.025% w/v sorbitol, and 0.025% v/v methanol) to confirm that each strain was able to growth under these conditions (Fig. S2b). To test the effect of a strain on the growth of Pe299R, Pe299R::mSc was mixed in a 1:1 ratio with a second strain and red fluorescence intensity was measured over time (Fig. 3a). If normalised by the fluorescence signal of a monoculture, constitutive fluorescence expression was shown to serve as a proxy for changes in bacterial biomass of individual strains in pairwise competitions [43].

Growth parameters from the fluorescence curves (μ_RFU_, K_RFU_, AUC_RFU_) were retrieved and compared with a competitive ability score (Eq. 1, Table S3). This competition score includes both μ_RFU_ and K_RFU_ in interspecific competition (Pe299R::mSc vs. *sp*_2_) in comparison to intraspecific competition (Pe299R::mSc vs Pe299R) and the monoculture (Pe299R::mSc). The competition score showed a strong correlation with most metrics (|*r* | > 0.96), except with estimated growth rates alone (Fig. S4, *r* = 0.67). Thus, changes in Pe299R growth as measured by the AUC of the fluorescent growth curve in relation to the monoculture can be explained by the ability of a strain to compete with Pe299R (Fig. 3b, *R*^2^ = 0.98, *F*_1,25_ = 1272, *p* < 0.05). The highest competition scores were observed for PssB728a, PkP19E3, and ArthL145, while the lowest were RhodL225, MethL85, and SmFR1. Regression analysis was used to evaluate the effect of MRO and/or phylogenetic distances in the competition scores against Pe299R (Table S4). These competition outcomes were partially explained by MRO_M5C_ (Fig. 3c, *R*^2^ = 0.46, *F*_1,22_ = 20.67, β = 13.04, *p* < 0.05, 95% CI [7.09, 18.99]). PssB728a showed the largest deviation from the regression model, suggesting that mechanisms other than competition for carbon could explain the increased competitive ability of PssB728a in vitro. However, no interference competition was observed in double-layer assays on R2A (Fig. S5). This is congruent with previous findings showing that interference competition is rare among phyllosphere bacteria [15]. A second linear regression in which the competition between Pe299R and PssB728a was excluded from the analysis showed that MRO_M5C_ explained more accurately the competitive ability of a strain against Pe299R (Table S4, *R*^2^ = 0.81, *F*_1,18_ = 83.45, β = 13.65, *p* < 0.05, 95% CI [10.51, 16.78]). Alternatively, a generalised linear model including MRO_M5C_, phylogenetic distance (PD), and the interaction between these terms explained the competitive ability of an epiphyte against Pe299R (Table S4, Gamma error distribution with a log link, *F*_1,20_ = 9.61, *p* = 0.0056). In this model, the competitive score of an epiphyte depends on the interaction between MRO_M5C_ and PD (MRO_M5C_×PD: *p* = 0.0062), in which the competitiveness of closely-related species to Pe299R are less dependent on MRO_M5C_ than distantly-related species (Fig. S6a). These results indicate that 89.5% of the variation in the in vitro competitiveness of an epiphyte against Pe299R in defined media can be explained by the utilised resources that they have in common (Null deviance = 7.60, Residual deviance = 0.801, pseudo-*R*^2^ = 0.89), as predicted by the MRO, and their phylogenetic distance.

### Bacterial competition in the phyllosphere at different scales reflect different competition outcomes

#### Competition at the population scale

The effect of a competitor on the growth of Pe299R was evaluated in the arabidopsis phyllosphere by co-inoculating four-week-old arabidopsis plants with Pe299R_CUSPER_ (Pe299R::mSc (pProbe_CUSPER)) and a second strain to estimate changes in population densities as well as single-cell reproductive success of Pe299R *in planta*.

**Fig. 4:**
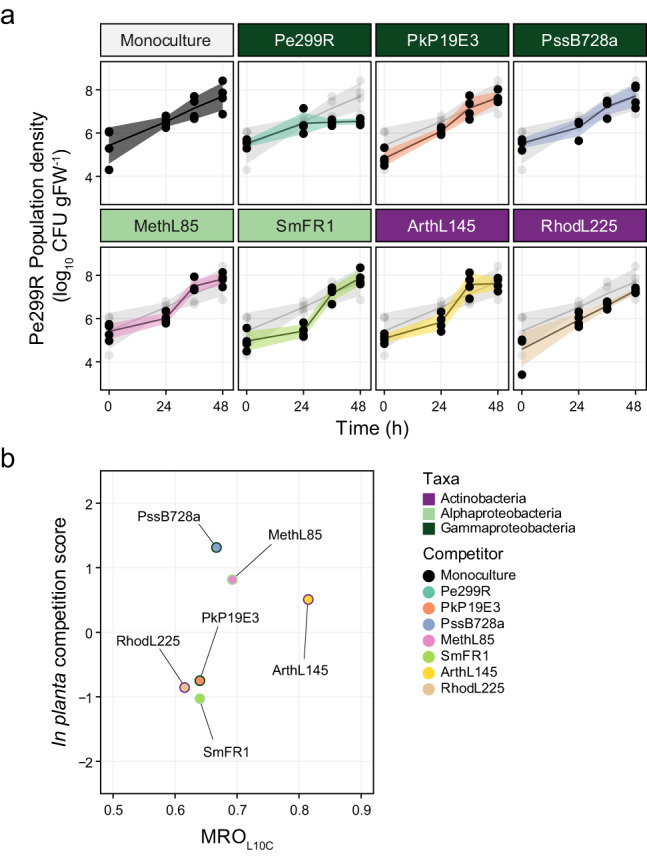
Changes of Pe299R population density in the phyllosphere. **a** Population size of *P. eucalypti* 299R::Tn*7*::mSc::Gm^R^(pProbe_CUSPER) (*Pe*299R_CUSPER_) on arabidopsis plants as monoculture or in the presence of a second epiphyte (top label). Each data point represents the CFU of Pe299R per gram of fresh leaf weight (CFU gFW^−1^) of individual plants (*n* = 4) at different sampling points (0, 24, 36, and 48 h). Groups were compared using two-way ANOVA, with a significance level of α = 0.05. For comparison, the monoculture curve is shown in every plot in light grey. **b** Relationship between resource overlap (MRO_L10C_) and the competition score of an epiphyte against Pe299R in the phyllosphere. Details of the regression model can be found in Table S5.

In every case, total bacterial density was determined and increased over time to a similar maximal load (Fig. S7). Particularly, changes in CFU of Pe299R were dependent on both sampling time and presence of a competitor (Time× Competitor: *F*_1,21_ = 3.34, *p* < 0.05). Compared to monoculture, the presence of SmFR1 only impacted initially (24 hpi) the Pe299R_CUSPER_ population, and the near-isogenic Pe299R at 48 hpi (Fig. 4a).

In an effort to explain differences in competitive abilities of the strains *in planta*, different MRO indexes were calculated based on carbon sources that have been detected on arabidopsis leaves (Fig. S8, Table 2). Despite the small impact detected on the Pe299R_CUSPER_ population at the CFU-level, the MRO with the most predictive power was the one calculated from a medium composition including ten carbon sources (MRO_L10C_): fumarate, sucrose, aspartate, malate, citrate, glutamate, alanine, fructose, threonine, and methanol (L10C, Table S1). These resources were the ten most abundant metabolites detected in arabidopsis leaves [39]. Particularly, compared to a similar composition including eight resources (L8C, Table S1), the presence of citrate, alanine, and threonine, as well as the absence of glucose, increased the predictability of competition outcomes, through an increase in the MRO between Pe299R and ArthL145 (MRO_L8C_ = 0.69; MRO_L10C_ = 0.81), and Pe299R and PssB728a (MRO_L8C_ = 0.59; MRO_L10C_ = 0.67). However, this effect was significant only when a linear regression model included MRO_L10C_ and the phylogenetic distance (PD) between an epiphyte and Pe299R (Fig. 4b, *R*^2^ = 0.92, *F* = 20.15, *p* = 0.048). The regression model suggests that the competitive ability of an epiphyte against Pe299R depends on both their resource overlap and phylogenetic relationships (Table S5, MRO_L10C_×PD: *p* = 0.029). In the phyllosphere, high competition scores were observed among closely related species with high MRO_L10C_ (Figs. 4b, S6b). In summary, Pe299R population density in the phyllosphere was not largely affected by the presence of a competitor, and differences in the competitive ability of this second strain could be explained by both its resource overlap and phylogenetic relationship with Pe299R.

#### Competition at the single cell-resolution

An improved version of the CUSPER bioreporter plasmid was constructed and was used to develop Pe299R_CUSPER_ (Fig. S9). In contrast to the initial CUSPER bioreporter, Pe299R_CUSPER_ constitutively expresses a red fluorescent protein and carries the recently developed green fluorescent protein mClover3 in a multicopy plasmid, rather than a chromosomally inserted single copy of GFPmut3. Pe299R_CUSPER_ is a bioreporter that estimates the reproductive success (RS) of immigrant cells in a new environment by back-calculating the number of divisions a cell underwent since its arrival [30–32].

The RS was determined by measuring the reduction in single cell green fluorescence compared to the mean green fluorescence of the population at time zero (*t*_0_), ex situ. Thereby, the reproductive success of a population and individual cells can be estimated. The limit of detection was determined based on the empirical cumulative distribution function from background fluorescence signals (Fig. S10a). A 5% probability represents RS values equal or greater than 4.58. Thus, a limit of detection of 4.5 cell divisions was selected (Fig. S10b). Cells with RS values above 4.5 were grouped and considered to undergo more than four divisions (RS_>4_).

The relative increase in Pe299R_CUSPER_ population from the initial inoculum at a given time of sampling can be estimated based on the fraction of cells in a particular subpopulation and the number of divisions that a cell is expected to undergo upon arrival in the phyllosphere [31]. The increase in Pe299R_CUSPER_ population from single-cell data was associated with the increase in population size at the CFU level (*R*^2^ = 0.63, *F*_1,382_ = 655.6, *p* < 0.05), suggesting that the single-cell measurements and the threshold used were adequate to assess changes in Pe299R_CUSPER_ populations. Similar to the results of the CFU-based population-level experiment above, the presence of competitors did not affect the average single-cell reproductive success of the Pe299R_CUSPER_ population compared to the monoculture (Fig. S11).

**Fig. 5:**
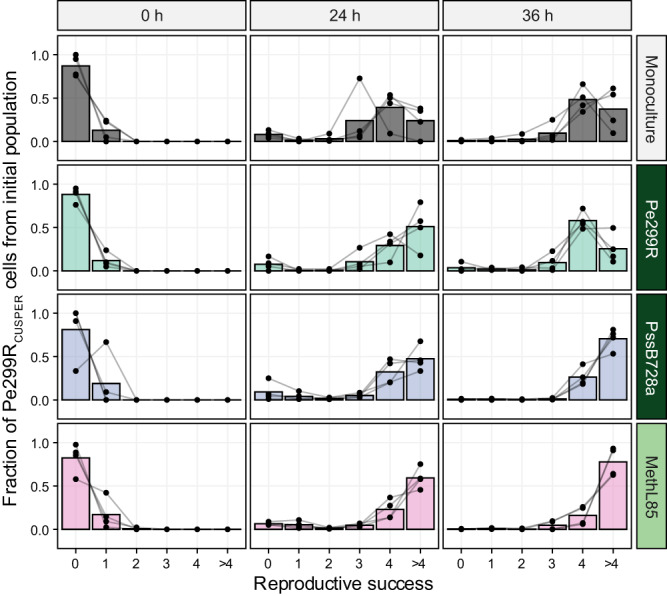
Changes in the composition of the Pe299R_CUSPER_ populations over time in the presence of a second epiphyte. Relative fraction of the reproductive success of the founder population to the observed Pe299R_CUSPER_ population at each sampling time point (0, 24, and 36 hpi) as monoculture or in the presence of a second epiphyte. The relative fractions of each biological replicate are shown and connected with a grey line. The bar represents the mean relative fraction for all replicates. Dark green label: gammaproteobacterial; light green label: alphaproteobacteria. The full version of this figure can be found in Fig. S13.

The distribution of immigrant cells that experienced different levels of reproductive success was analysed as relative fractions of the initial population. Cell groups were binned based on the number of divisions that the respective ancestral immigrant cell underwent after inoculation. The different bins for the number of divisions –the reproductive success– ranged from 0 to >4 generations after inoculation. Consequently, a population structure of Pe299R_CUSPER_ was defined based on the relative fraction of cells with different RS. A 36.94% of the variation in the population structure of Pe299R_CUSPER_ could be explained by the time of sampling (*R*^2^ = 0.10, *F*_1,55_ = 8.69, *p* = 0.001) and the presence of competitors (*R*^2^ = 0.27, *F*_7,55_ = 3.36, *p* = 0.002) using PERMANOVA (Table S6). The initial population was composed of cells with zero (RS_0_) or one division (RS_1_) across the different treatments, and were excluded from the multivariate analysis, as it only accounts for the initial population and not for underlying competitive interactions. At 24 and 36 hpi, cells that divided three and more times contributed most to the final populations, whose distribution became bimodal (Fig. S12). For Pe299R_CUSPER_ in the presence of Pk19E3, PssB728a, ArthL145, RhodL225, and MethyL85, a relatively high fraction of cells that divide between zero and three times was observed at 24 hpi (Fig. S13). However, only the presence of PssB728a (*R*^2^ = 0.24, *F*_1,14_ = 4.50, *p* = 0.011) and MethL85 (*R*^2^ = 0.36, *F*_1,14_ = 7.80, *p* = 0.003) led to a differentiation in the population structure of Pe299R_CUSPER_ compared to the monoculture (Figs. 5, S14). Compared to Pe299R_CUSPER_ as monoculture, the presence of PssB728a and MethL85 increased the relative fraction of Pe299R_CUSPER_ cells that divided more than four times. As PssB728a and MethL85 exhibited an MRO_L10C_ with Pe299R of 0.67 and 0.69, respectively, which lie within one standard deviation from the mean MRO_L10C_ of the tested strains (0.68 ± 0.072), the effect of epiphytes on the structure of the Pe299R population in the phyllosphere cannot be associated solely with their resource overlaps.

## Discussion

Understanding microbial community structure and dynamics in the phyllosphere requires a deeper investigation into the mechanisms that influence local microbe-microbe interactions. As resources are a limiting factor for bacterial colonisation [53], and negative interactions are common outcomes between microbes in the phyllosphere [14], we hypothesised that resource competition is the dominant type of interaction in this environment. A negative correlation between coexistence and similarity in resource utilisation has been shown for a number of pairs of epiphytic bacteria, including the focal species Pe299R [17]. On arabidopsis, plant-protective *Sphingomonas* spp. decrease the population of the phytopathogen *Pseudomonas syringae* pv. *tomato* DC3000. Although some sphingomonads suppress the proliferation of *P. syringae* pv. *tomato* DC3000 via priming of the plant immunity [54], the high resource overlaps between *P. syringae* pv. *tomato* DC3000 and the *Sphingomonas* spp. suggest that resource competition explains in parts the decrease in population size of the pathogen [55]. However, estimating bacterial resource preferences in complex and heterogeneous environments is challenging. In this work, genome-scale metabolic modelling was used to predict the outcome of species interactions under homogeneous in vitro conditions as well as in the heterogeneous phyllosphere.

Similarity in resource utilisation has been used to define a niche overlap index that includes a wide range of carbon sources, many of which are unlikely to be relevant on the leaf surface [17]. The availability of resources in a given environment and resource preference of competitors determine the effective resource overlap [56]. MRO is an index that incorporates the minimal growth requirements of a species’ metabolic model under defined media composition in silico [22]. To our knowledge, MRO has not been used in combination or validated with controlled empirical studies. For this reason, we validated the use of MRO based on strains that could metabolise at least one of five different carbon sources (glucose, fructose, sorbitol, fumarate, and methanol). Our results show that MRO reflects the dissimilarities in resource utilisation in bacterial batch cultures. The strong correlation between MRO and resource utilisation dissimilarity was mainly explained by similarities in estimated μ. This growth parameter was determined at 24 h intervals, which might be inaccurate for fast-growing bacteria such as Pe299R. However, no significant differences were observed in growth rates of Pe299R when curves with high temporal resolution were fitted into a logistic growth curve (Fig. S1).

To test whether MRO also predicts competition outcomes in heterogeneous environments, the similarity of resource preferences was selected to link competitive differences among epiphytes on leaves. Similar to other natural environments, the resource landscape on leaves is uneven and otherwise challenging to measure [1, 2, 57, 58]. The MRO calculated from the ten most abundant resources in the arabidopsis leaf metabolome (MRO_L10C_) [39] were partially predictive for the competition outcomes of the here-studied strains, as MRO was only significant in combination with phylogenetic distances, explaining 53% of the variation. It is worth noting that the selected resources cannot be generalised for competitions on leaves, as other strains could compete for resources that were not included in the MRO calculation, such as low abundant or rare resources [59], vitamins [60], or iron [57, 61]. Additionally, the algorithm to calculate MRO does not include the concentration of resources a priori, which could be a limitation in our estimations, especially in the phyllosphere. However, MRO calculated using additional resources did not increase the explanatory power of resource overlap in this study, suggesting that major competition in the phyllosphere is restricted to a limited set of most abundant resources.

Generally, predictability of the competition ability in vitro and, especially in the phyllosphere improved when phylogenetic distances were accounted for as a factor in the analysis. Phylogenetic distance was included in the model, as taxon-dependent characteristics may favour either high or low phylogenetic diversity [62]. For example, competition between *Pseudomonas fluorescens* SBW25 and other species decreased at increasing phylogenetic distances, which correlated with increased niche differences [63]. In vitro, we observed that the competition of species that were phylogenetically closely related with Pe299R, PssB728a and PkP19E3, was less dependent on MRO than other strains. This suggests that conserved traits rather than carbon utilisation could be more relevant in the competition among the tested bacterial pairs. Since the family *Pseudomonadaceae* exhibits the greatest functional diversity among plant-associated bacteria [64], there may be additional mechanisms that could increase the competitiveness of *Pseudomonas* spp. against Pe299R such as the production of inhibitory compounds [15]. Although our results indicate that there was no direct inhibition between PssB728a and PkP19E3 on R2A medium (Fig. S4), the expression of type VI secretion systems and toxins can be context-dependent, as shown for *Pseudomonas syringae* pv. *tomato* DC3000 [65, 66].

We observed that MRO does not correlate with phylogenetic distances, which is in agreement with previous findings showing that genes associated with carbon source utilisation are not phylogenetically conserved in bacteria [67]. The lack of phylogenetic conservation also applies to plant-associated bacteria, as comparative genomic analysis of the arabidopsis microbiota showed a high overlap of genes linked to carbon and amino acid metabolism, independent of their phylogeny [64]. The results presented in this work suggest that not only resource competition, but evolutionary-conserved traits contribute to competition outcomes in the phyllosphere. Traits such as aggregation, motility, communication, osmotolerance, and production of secondary metabolites such as inhibitory compounds, biosurfactants, and/or siderophores could influence the fitness of leaf colonisers. Our results are therefore congruent with modern coexistence theory, where competitive exclusion depends on niche differences (e.g., high resource overlap) and fitness differences between pairs of species [12, 68]. However, in the presence of multiple competitors, it remains a challenge to address the effect of multitrophic interaction networks on species coexistence. Hence, pairwise competition outcomes may fail to predict community dynamics; instead, mechanisms such as intransitive loops or higher-order interactions may better explain community diversity of a community [69, 70].

Pe299R_CUSPER_ was used to measure the single-cell reproductive success of Pe299R and to estimate bacterial fitness in competition *in planta*. This bioreporter relies on the fluorescence intensity of individual cells, which can be traced back to the dilution of a fluorescent protein after cell division [30]. The number of divisions that can be determined is however limited to the initial four cell divisions. This bioreporter was instrumental in understanding that bacterial populations in the phyllosphere separate into subpopulations over time [30].

The observed RS heterogeneity within arabidopsis-colonising Pe299R_CUSPER_ is congruent with findings in the phyllosphere of bush bean leaves (*Phaseolus vulgaris*) [30]. This supports the notion that variable habitability is a common feature of the phyllosphere of different species. The plant host impacts on bacterial colonisation, suggesting that the host could influence bacteria-bacteria interactions by environment modifications through variations of metabolite availability during the circadian cycle [38], leaf side [71], leaf development [72, 73], ageing [74], and cuticle composition [75]. Differences in reproductive success within the Pe299R population correlate with, but are not limited to, spatially distinct resource pools, such as carbohydrates and water, on leaves [1, 2, 76]. Considering the variable fate of bacterial cells during leaf colonisation, the effect of resource overlap in bacterial interactions was expected to be evaluated at the single-cell level. While we showed that the presence of other strains did not lead to differences compared to the Pe299R_CUSPER_ monoculture, PssB728a and MethL85 positively affected RS at the single cell level.

Despite showing the highest population-level competitive scores while not featuring a notably low MRO, the fraction of successful Pe299R cells (>4 divisions) were higher in the presence of PssB728a compared to the monoculture. This suggests that mechanisms other than resource competition were influencing the interactions between Pe299R and PssB728a. The pseudomonad PkP19E shows a similar, albeit not statistically significant, effect by increasing the fraction of cells in the Pe299R population that has a RS > 4. *Pseudomonas* spp. produce biosurfactants, i.e. amphiphilic molecules that decrease water surface tension and thereby increase resource permeability onto the leaf surface, increasing bacterial survival due to their water retaining hygroscopic nature [77–80]. By producing biosurfactants, *Pseudomonas* spp. could thereby benefit Pe299R. Alternatively, these strains could engage in cooperative interactions such as cross-feeding, as observed between *Pantoea* spp. and *Pseudomonas koreensis* in the *Flaveria robusta* leaf apoplast [81]. However, further investigations are required to understand the mechanisms that result in beneficial interactions in the phyllosphere.

MethL85 belongs to a group of resource specialists and facultative methylotrophs from the genus *Methylobacterium*. Methanol utilisation is a fitness advantage in the phyllosphere, as methylotrophs can utilise the released methanol from the plant cell wall metabolism [40]. As one carbon metabolism is highly overrepresented in proteomes of methylobacteria on leaves [82, 83], it is expected that MethL85 utilises methanol as a main carbon source and does not compete with Pe299R for their preferred carbon sources. Although there is no evidence yet of mutualism between methylobacteria and other taxa in natural conditions, the biosurfactant production of MethL85 may act as a water retaining factor, supporting the spread of bacteria [77, 78]. Alternatively, dead MethL85 biomass could increase the survival of Pe299R [84]. Hence, additional growth of Pe299R in presence of MethL85 compared to a near isogenic co-inoculant is not unexpected.

Our results suggest that there is little impact of resource overlap on the competition between bacteria that co-colonise the leaf surface. This could be a result of the strong segregation of habitable sites on leaves and low initial bacterial densities at the time of inoculation. Thus, resource competition in combination with historical contingency caused by priority effects could have a larger impact on competition outcomes. Although co-colonisation had little effect on Pe299R, this could change after pre-emptive colonisation by a competitor [32, 61].

Overall, we observed a relationship between the resource overlap and the competition of pairs of species in both homogeneous and heterogeneous environments. This relationship was stronger and more predictive in vitro compared to the phyllosphere. However, single-cell measurements did not correlate with population-level measurements, indicating that competition is operating at the micrometre, or single-cell resolution and thus, local competition cannot be investigated by measuring interactions and changes in population densities at the whole-leaf scale. Regardless, our findings support an important role of resource overlap in community assembly processes of bacteria in the phyllosphere. This is in line with previous findings that related resource overlap of competitors with disease severity on tomato plants [85] and co-existence of near-isogenic strains that differed only in the ability to metabolise an additional resource [17].

Understanding the impact of resource competition during bacterial community assemblage in the phyllosphere has major implications in developing effective biocontrol strategies against phytopathogens [86, 87]. Many bacterial foliar pathogens undergo an epiphytic phase during the initial colonisation of leaves [88]. This phase is characterised by population growth before invading the endophytic compartments. The rational design of biocontrol agents or communities to reduce pathogen populations in the phyllosphere through competitive interactions could prevent crop losses caused by microbial diseases. However, our findings showed that colonisation prevention of leaves by bacteria that feature different degrees of resource overlap in the phyllosphere is challenging. Previous metrics of resource overlap that considered many different resources are not the best strategy to select for strong competitors against a focal species [17]. Instead, resource overlap metrics should consider resources that are most relevant in the system and for the phytopathogen to be controlled, and traits that are phylogenetically conserved. In this study, ten resources detected in arabidopsis leaves had the most predictive power. Additional resources did not increase the predictive outcome of the metric (Table S5). Thus, we consider MRO in conjunction with information of resource abundances in the phyllosphere of arabidopsis more suitable than previous metrics.

## Supplementary information

Supplemental Material

## Data Availability

Metabolic models, data, and R codes used for statistical analyses are available on the GitHub repository https://github.com/roschlec/paper_cusper_competition.

## References

[1] Doan HK, Ngassam VN, Gilmore SF, Tecon R, Parikh AN, Leveau JHJ (2020). Topography-driven shape, spread, and retention of leaf surface water impacts microbial dispersion and activity in the phyllosphere. Phytobiomes J.

[2] Leveau JH, Lindow SE (2001). Appetite of an epiphyte: quantitative monitoring of bacterial sugar consumption in the phyllosphere. Proc Natl Acad Sci USA.

[3] Remus-Emsermann MNP, Schlechter RO (2018). Phyllosphere microbiology: at the interface between microbial individuals and the plant host. N Phytol.

[4] Remus-Emsermann MNP, Lücker S, Müller DB, Potthoff E, Daims H, Vorholt JA (2014). Spatial distribution analyses of natural phyllosphere-colonizing bacteria on *Arabidopsis thaliana* revealed by fluorescence in situ hybridization. Environ Microbiol.

[5] Esser DS, Leveau JHJ, Meyer KM, Wiegand K (2015). Spatial scales of interactions among bacteria and between bacteria and the leaf surface. FEMS Microbiol Ecol.

[6] Steinberg S, Grinberg M, Beitelman M, Peixoto J, Orevi T, Kashtan N (2021). Two-way microscale interactions between immigrant bacteria and plant leaf microbiota as revealed by live imaging. ISME J.

[7] Schlechter RO, Miebach M, Remus-Emsermann MNP (2019). Driving factors of epiphytic bacterial communities: A review. J Adv Res.

[8] Agler MT, Ruhe J, Kroll S, Morhenn C, Kim S-T, Weigel D (2016). Microbial hub taxa link host and abiotic factors to plant microbiome variation. PLoS Biol.

[9] Carlström CI, Field CM, Bortfeld-Miller M, Müller B, Sunagawa S, Vorholt JA (2019). Synthetic microbiota reveal priority effects and keystone strains in the *Arabidopsis* phyllosphere. Nat Ecol Evol.

[10] Lidicker WZ (1979). A clarification of interactions in ecological systems. Bioscience.

[11] Hassani MA, Durán P, Hacquard S (2018). Microbial interactions within the plant holobiont. Microbiome.

[12] Chesson P (2000). Mechanisms of maintenance of species diversity. Annu Rev Ecol Syst.

[13] Letten AD, Ke P-J, Fukami T (2017). Linking modern coexistence theory and contemporary niche theory. Ecol Monogr.

[14] Schäfer M, Vogel CM, Bortfeld-Miller M, Mittelviefhaus M, Vorholt JA (2022). Mapping phyllosphere microbiota interactions *in planta* to establish genotype-phenotype relationships. Nat Microbiol.

[15] Helfrich EJN, Vogel CM, Ueoka R, Schäfer M, Ryffel F, Müller DB (2018). Bipartite interactions, antibiotic production and biosynthetic potential of the *Arabidopsis* leaf microbiome. Nat Microbiol.

[16] Wilson M, Lindow SE (1994). Ecological similarity and coexistence of epiphytic ice-nucleating (Ice^+^) *Pseudomonas syringae* strains and a non-ice-nucleating (Ice^−^) biological control agent. Appl Environ Microbiol.

[17] Wilson M, Lindow SE (1994). Coexistence among epiphytic bacterial populations mediated through nutritional resource partitioning. Appl Environ Microbiol.

[18] Dianese AC, Ji P, Wilson M (2003). Nutritional similarity between leaf-associated nonpathogenic bacteria and the pathogen is not predictive of efficacy in biological control of bacterial spot of tomato. Appl Environ Microbiol.

[19] Diener C, Gibbons SM, Resendis-Antonio O (2020). MICOM: Metagenome-scale modeling to infer metabolic interactions in the gut microbiota. mSystems.

[20] Muller EEL, Faust K, Widder S, Herold M, Arbas SM, Wilmes P (2018). Using metabolic networks to resolve ecological properties of microbiomes. Curr Opin Syst Biol.

[21] Freilich S, Zarecki R, Eilam O, Segal ES, Henry CS, Kupiec M (2011). Competitive and cooperative metabolic interactions in bacterial communities. Nat Commun.

[22] Zelezniak A, Andrejev S, Ponomarova O, Mende DR, Bork P, Patil KR (2015). Metabolic dependencies drive species co-occurrence in diverse microbial communities. Proc Natl Acad Sci USA.

[23] Hester ER, Jetten MSM, Welte CU, Lücker S (2019). Metabolic overlap in environmentally diverse microbial communities. Front Genet.

[24] Dal Co A, van Vliet S, Kiviet DJ, Schlegel S, Ackermann M (2020). Short-range interactions govern the dynamics and functions of microbial communities. Nat Ecol Evol.

[25] Tecon R, Ebrahimi A, Kleyer H, Levi SE, Or D (2018). Cell-to-cell bacterial interactions promoted by drier conditions on soil surfaces. Proc Natl Acad Sci USA.

[26] Monier J-M, Lindow SE (2005). Aggregates of resident bacteria facilitate survival of immigrant bacteria on leaf surfaces. Micro Ecol.

[27] Wilson M, Hirano SS, Lindow SE (1999). Location and survival of leaf-associated bacteria in relation to pathogenicity and potential for growth within the leaf. Appl Environ Microbiol.

[28] Tecon R, Leveau JHJ (2012). The mechanics of bacterial cluster formation on plant leaf surfaces as revealed by bioreporter technology. Environ Microbiol.

[29] Remus-Emsermann MNP, Kim EB, Marco ML, Tecon R, Leveau JHJ (2013). Draft genome sequence of the phyllosphere model bacterium *Pantoea agglomerans* 299R. Genome Announc.

[30] Remus-Emsermann MNP, Leveau JHJ (2010). Linking environmental heterogeneity and reproductive success at single-cell resolution. ISME J.

[31] Remus-Emsermann MNP, Tecon R, Kowalchuk GA, Leveau JHJ (2012). Variation in local carrying capacity and the individual fate of bacterial colonizers in the phyllosphere. ISME J.

[32] Remus-Emsermann MNP, Kowalchuk GA, Leveau JHJ (2013). Single-cell versus population-level reproductive success of bacterial immigrants to pre-colonized leaf surfaces. Environ Microbiol Rep.

[33] Peyraud R, Kiefer P, Christen P, Massou S, Portais J-C, Vorholt JA (2009). Demonstration of the ethylmalonyl-CoA pathway by using ^13^C metabolomics. Proc Natl Acad Sci USA.

[34] Wu M, Eisen JA (2008). A simple, fast, and accurate method of phylogenomic inference. Genome Biol.

[35] Paradis E, Schliep K (2019). ape 5.0: an environment for modern phylogenetics and evolutionary analyses in R. Bioinformatics.

[36] Sprouffske K, Wagner A (2016). Growthcurver: an R package for obtaining interpretable metrics from microbial growth curves. BMC Bioinforma.

[37] Machado D, Andrejev S, Tramontano M, Patil KR (2018). Fast automated reconstruction of genome-scale metabolic models for microbial species and communities. Nucl Acids Res.

[38] Augustijn D, Roy U, van Schadewijk R, de Groot HJM, Alia A (2016). Metabolic profiling of intact *Arabidopsis thaliana* leaves during circadian cycle using ^1^H high resolution magic angle spinning NMR. PLoS One.

[39] Badri DV, Zolla G, Bakker MG, Manter DK, Vivanco JM (2013). Potential impact of soil microbiomes on the leaf metabolome and on herbivore feeding behavior. N Phytol.

[40] Corpe WA, Rheem S (1989). Ecology of the methylotrophic bacteria on living leaf surfaces. FEMS Microbiol Lett.

[41] Dorokhov YL, Sheshukova EV, Komarova TV (2018). Methanol in plant life. Front Plant Sci.

[42] Bennett L, Melchers B, Proppe B Curta: A General-purpose high-performance computer at ZEDAT, Freie Universität Berlin. 2020.

[43] Schlechter RO, Kear EJ, Remus DM, Remus-Emsermann MNP (2021). Fluorescent protein expression as a proxy for bacterial fitness in a high-throughput assay. Appl Environ Microbiol.

[44] Hart SP, Freckleton RP, Levine JM (2018). How to quantify competitive ability. J Ecol.

[45] Godwin CM, Chang F-H, Cardinale BJ (2020). An empiricist’s guide to modern coexistence theory for competitive communities. Oikos.

[46] Miebach M, Schlechter RO, Clemens J, Jameson PE, Remus-Emsermann MNP (2020). Litterbox—A gnotobiotic zeolite-clay system to investigate arabidopsis-microbe interactions. Microorganisms.

[47] Schindelin J, Arganda-Carreras I, Frise E, Kaynig V, Longair M, Pietzsch T (2012). Fiji: an open-source platform for biological-image analysis. Nat Methods.

[48] Dixon P (2003). VEGAN, a package of R functions for community ecology. J Veg Sci.

[49] R Core Team. R: A language and environment for statistical computing. *R Foundation for Statistical Computing, Vienna, Austria*. https://www.R-project.org/.

[50] Wickham H, Averick M, Bryan J, Chang W, McGowan LD, François R (2019). Welcome to the Tidyverse. J Open Source Softw.

[51] Gu Z, Eils R, Schlesner M (2016). Complex heatmaps reveal patterns and correlations in multidimensional genomic data. Bioinformatics.

[52] Navarro D. Learning statistics with R: A tutorial for psychology students and other beginners. (Version 0.6). University of New South Wales, Sydney, Australia. R package version 0.5.1, 2015. https://learningstatisticswithr.com.

[53] Mercier J, Lindow SE (2000). Role of leaf surface sugars in colonization of plants by bacterial epiphytes. Appl Environ Microbiol.

[54] Vogel CM, Potthoff DB, Schäfer M, Barandun N, Vorholt JA (2021). Protective role of the *Arabidopsis* leaf microbiota against a bacterial pathogen. Nat Microbiol.

[55] Innerebner G, Knief C, Vorholt JA (2011). Protection of *Arabidopsis thaliana* against leaf-pathogenic *Pseudomonas syringae* by *Sphingomonas* strains in a controlled model system. Appl Environ Microbiol.

[56] May RM, MacArthur RH (1972). Niche overlap as a function of environmental variability. Proc Natl Acad Sci USA.

[57] Loper JE, Lindow SE (1994). A biological sensor for iron available to bacteria in their habitats on plant surfaces. Appl Environ Microbiol.

[58] Hernandez MN, Lindow SE (2019). *Pseudomonas syringae* increases water availability in leaf microenvironments via production of hygroscopic syringafactin. Appl Environ Microbiol.

[59] Hemmerle L, Ochsner AM, Vonderach T, Hattendorf B, Vorholt JA (2021). Mass spectrometry-based approaches to study lanthanides and lanthanide-dependent proteins in the phyllosphere. Methods Enzymol.

[60] Ryback B, Bortfeld-Miller M, Vorholt JA (2022). Metabolic adaptation to vitamin auxotrophy by leaf-associated bacteria. ISME J.

[61] Müller L, Müller DC, Kammerecker S, Fluri M, Neutsch L, Remus Emsermann M (2022). Priority effects in the apple flower determine if the siderophore desferrioxamine is a virulence factor for *Erwinia amylovora* CFBP1430. Appl Environ Microbiol.

[62] Mayfield MM, Levine JM (2010). Opposing effects of competitive exclusion on the phylogenetic structure of communities. Ecol Lett.

[63] Tan J, Slattery MR, Yang X, Jiang L (2016). Phylogenetic context determines the role of competition in adaptive radiation. Proc Biol Sci.

[64] Bai Y, Müller DB, Srinivas G, Garrido-Oter R, Potthoff E, Rott M (2015). Functional overlap of the *Arabidopsis* leaf and root microbiota. Nature.

[65] Haapalainen M, Mosorin H, Dorati F, Wu R-F, Roine E, Taira S (2012). Hcp2, a secreted protein of the phytopathogen *Pseudomonas syringae* pv. *tomato* DC3000, is required for fitness for competition against bacteria and yeasts. J Bacteriol.

[66] Chien C-F, Liu C-Y, Lu Y-Y, Sung Y-H, Chen K-Y, Lin N-C (2020). HSI-II gene cluster of *Pseudomonas syringae* pv. *tomato* DC3000 encodes a functional type VI secretion system required for interbacterial competition. Front Microbiol.

[67] Martiny AC, Treseder K, Pusch G (2013). Phylogenetic conservatism of functional traits in microorganisms. ISME J.

[68] HilleRisLambers J, Adler PB, Harpole WS, Levine JM, Mayfield MM (2012). Rethinking community assembly through the lens of coexistence theory. Annu Rev Ecol Evol Syst.

[69] Levine JM, Bascompte J, Adler PB, Allesina S (2017). Beyond pairwise mechanisms of species coexistence in complex communities. Nature.

[70] Gallien L, Zimmermann NE, Levine JM, Adler PB (2017). The effects of intransitive competition on coexistence. Ecol Lett.

[71] Smets W, Chock MK, Walsh CM, Vanderburgh CQ, Kau E, Lindow SE, et al. Leaf side determines the relative importance of dispersal versus host filtering in the phyllosphere microbiome. *bioRxiv* 2022; 2022.08.16.504148: 10.1101/2022.08.16.504148. 10.1128/mbio.01111-23 PMC10470611 37436063

[72] Copeland JK, Yuan L, Layeghifard M, Wang PW, Guttman DS (2015). Seasonal community succession of the phyllosphere microbiome. Mol Plant Microbe Interact.

[73] Beilsmith K, Perisin M, Bergelson J (2021). Natural bacterial assemblages in *Arabidopsis thaliana* tissues become more distinguishable and diverse during host development. mBio.

[74] Wagner MR, Lundberg DS, Del Rio TG, Tringe SG, Dangl JL, Mitchell-Olds T (2016). Host genotype and age shape the leaf and root microbiomes of a wild perennial plant. Nat Commun.

[75] Bodenhausen N, Bortfeld-Miller M, Ackermann M, Vorholt JA (2014). A synthetic community approach reveals plant genotypes affecting the phyllosphere microbiota. PLoS Genet.

[76] Remus-Emsermann MNP, de Oliveira S, Schreiber L, Leveau JHJ (2011). Quantification of lateral heterogeneity in carbohydrate permeability of isolated plant leaf cuticles. Front Microbiol.

[77] Burch AY, Zeisler V, Yokota K, Schreiber L, Lindow SE (2014). The hygroscopic biosurfactant syringafactin produced by *Pseudomonas syringae* enhances fitness on leaf surfaces during fluctuating humidity. Environ Microbiol.

[78] Oso S, Walters M, Schlechter RO, Remus-Emsermann MNP (2019). Utilisation of hydrocarbons and production of surfactants by bacteria isolated from plant leaf surfaces. FEMS Microbiol Lett.

[79] Schreiber L, Krimm U, Knoll D, Sayed M, Auling G, Kroppenstedt RM (2005). Plant-microbe interactions: identification of epiphytic bacteria and their ability to alter leaf surface permeability. N. Phytol.

[80] Oso S, Fuchs F, Übermuth C, Zander L, Daunaraviciute S, Remus DM, et al. Biosurfactants produced by phyllosphere-colonizing pseudomonads impact diesel degradation but not colonization of leaves of gnotobiotic *Arabidopsis thaliana*. Appl Environ Microbiol. 2021;87:e00091–21. 10.1128/AEM.00091-21 PMC8091026 33608298

[81] Murillo-Roos M, Abdullah HSM, Debbar M, Ueberschaar N, Agler MT (2022). Cross-feeding niches among commensal leaf bacteria are shaped by the interaction of strain-level diversity and resource availability. ISME J.

[82] Delmotte N, Knief C, Chaffron S, Innerebner G, Roschitzki B, Schlapbach R (2009). Community proteogenomics reveals insights into the physiology of phyllosphere bacteria. Proc Natl Acad Sci.

[83] Müller DB, Schubert OT, Röst H, Aebersold R, Vorholt JA (2016). Systems-level proteomics of two ubiquitous leaf commensals reveals complementary adaptive traits for phyllosphere colonization. Mol Cell Proteom.

[84] Wilson M, Lindow SE (1994). Inoculum density-dependent mortality and colonization of the phyllosphere by *Pseudomonas syringae*. Appl Environ Microbiol.

[85] Ji P, Wilson M (2002). Assessment of the importance of similarity in carbon source utilization profiles between the biological control agent and the pathogen in biological control of bacterial speck of tomato. Appl Environ Microbiol.

[86] Wei Z, Yang T, Friman V-P, Xu Y, Shen Q, Jousset A (2015). Trophic network architecture of root-associated bacterial communities determines pathogen invasion and plant health. Nat Commun.

[87] Vorholt JA, Vogel C, Carlström CI, Müller DB (2017). Establishing causality: opportunities of synthetic communities for plant microbiome research. Cell Host Microbe.

[88] Hirano SS, Upper CD (2000). Bacteria in the leaf ecosystem with emphasis on *Pseudomonas syringae*-a pathogen, ice nucleus, and epiphyte. Microbiol Mol Biol Rev.

