# Supplementary material for: Metabolic resource overlap impacts competition among phyllosphere bacteria
Source: ISME J. 2023 Jun 24;17(9):1445–54. doi: 10.1038/s41396-023-01459-0 (PMC10432529; doi:10.1038/s41396-023-01459-0)
Supplement: Supplementary file 1 — Supplemental Material [file 41396_2023_1459_MOESM1_ESM.pdf]

Supplementary Material for

Metabolic resource overlap impacts competition among phyllosphere bacteria

Rudolf O. Schlechter[1,2,3,4*], Evan J. Kear[2], Michał Bernach[1,2,3,5], Daniela M. Remus[6], and Mitja N. P. Remus-Emsermann[1,2,3,4*]

[1] Institute of Microbiology and Dahlem Centre of Plant Sciences, Department of Biology, Chemistry, Pharmacy, Freie Universität Berlin, Berlin, Germany.

[2] School of Biological Sciences, University of Canterbury, Christchurch 8011, New Zealand.

[3] Biomolecular Interaction Centre, University of Canterbury, Christchurch 8011, New Zealand.

[4] Bioprotection Research Core, University of Canterbury, Christchurch 8011, New Zealand.

[5] Department of Electrical and Computer Engineering, University of Canterbury, Christchurch 8011, New Zealand.

[6] Protein Science and Engineering, Callaghan Innovation, School of Biological Sciences, University of Canterbury, Christchurch, New Zealand.

***Corresponding authors:** Rudolf O. Schlechter, r.schlechter.jahn@fu-berlin.de and Mitja N. P. Remus-Emsermann, m.remus-emsermann@fu-berlin.de

Keywords: Bacterial fitness, Leaf surface, Epiphytes, Community assembly, Single cells, Plant-microbe interactions, Reproductive success

Preprint Servers: biorxiv

Author Contributions: R.O.S. and M.N.P.R-E. designed research; R.O.S., E.J.K., M.B., D.M.R. performed research; R.O.S analysed data; and R.O.S., and M.N.P.R-E. wrote the paper.

Competing Interest Statement: The authors declare no conflict of interest.

This file includes:

Extended Materials and Methods

Supplemental Figures S1 to S15

Supplemental Tables S1 to S7

References

Extended Materials and Methods

Cloning of pProbe_CUSPER

The plasmid pProbe_CUSPER (Fig. S8) was constructed via isothermal assembly [1]. To that end, DNA fragments were obtained through polymerase-chain reaction (PCR) using Phusion High-Fidelity DNA polymerase (Thermo Scientific), following the manufacturer's recommendations. Annealing temperatures (T_a) were chosen based on the respective melting temperature (t_m) of the primers (Table S7). Touchdown PCRs were performed to amplify PCR products with overlapping ends for isothermal assemblies, as described elsewhere [2]. The *lac* promoter $P_{A1/04/03}$ was amplified using as template the plasmid miniTn7(Gm)PA1/04/03-eyfp-a [3], which was a gift from Tim Tolker-Nielsen (Addgene plasmid # 111620), and the primers Plac_fw and Plac_rv (Table S7). The green fluorescent protein gene, *mClover3*, was amplified from the plasmid pMRE147 [2] using the primers fp_fw and fp_rv. The repressor element *lacI^q^* was amplified from the plasmid pCPP39 using the primers lacIq_fw and lacIq_rv. Finally, the gentamicin resistant gene *gmR* was amplified from the plasmid pMRE143 using the primers gmR_fw and gmR_rv. Following amplification, PCR fragments were purified using Monarch PCR clean up kit (New England Biolabs, Place). The plasmid pFru97 [4] was used for cloning of the pProbe_CUSPER backbone by restriction digest using HindIII and ScaI, which includes ori*V*, *rep*, *mob*, a terminator region and the *nptII* gene as selection marker. Fragments were consolidated into pProbe_CUSPER and used to transform Stellar™ Competent Cells (*E. coli* HST08, Takara Bio Inc., Japan).

Development of the *Pe*299R$_{CUSPER}$ bioreporter

The constitutively red fluorescent protein-expressing strain *Pantoea eucalypti* 299R::Tn7::mScarlet-I::Gm^{R} (Pe299R::mSc) [2] was transformed with pProbe_CUSPER (Fig. S8) through electroporation to obtain the new bioreporter *Pe299R*::Tn*7*::mScarlet-I::Gm^{R}(pProbe_CUSPER), from here onwards referred to as Pe299R$_{CUSPER}$. Pe299R$_{CUSPER}$ is an updated version of the CUSPER bioreporter [5], in that every genetic material is harboured in the plasmid pProbe_CUSPER and that it encodes the fluorescent protein mClover3, a brighter version than GFPmut3. Electrocompetent cells were prepared based on Gonzales *et al.* (2013) [6] with modifications. Overnight culture of Pe299R::mSc grown on nutrient agar (NA, HiMedia, India) supplemented with 15 µg mL^{-1} gentamicin was scraped off with a loop and resuspended in phosphate buffer saline (PBS, 0.2 g L^{-1} NaCl, 1.44 g L^{-1} Na_2HPO_4 and 0.24 g L^{-1} KH_2PO_4). The bacterial suspension was washed in cold 10% v/v glycerol twice by centrifugation (5 minutes at 5,000 × *g*). Finally, cells were resuspended in 100 µL 10% v/v glycerol and transferred into an ice cold 2-mm electroporation cuvette. For electroporation, 100 ng of plasmid pProbe_CUSPER were added into an ice cold 2-mm cuvette (Genesee Scientific) and mixed carefully. One pulse of 1.8 kV at 200 Ω and 25 µF was applied, then 900 µL of nutrient broth (NB) was quickly added and the mix was transferred to a 15-mL conical tube. Cells were incubated for 2 h at 30 °C with shaking (200 rpm), and then plated onto NA supplemented with 50 µg mL^{-1} kanamycin (Km). Pe299R$_{CUSPER}$ was routinely grown on NA with Km at 30°C.

IPTG optimisation

Expression of mClover3 in pProbe_CUSPER is under the control of the *lac* promoter P$_{A1/04/03}$ and repressed by LacI^{q}, which can be inhibited by the addition of isopropyl beta-D-1-thiogalactopyranoside (IPTG). To determine the optimal concentration of IPTG for mClover3 expression, fluorescence was measured at different concentrations of IPTG in a FLUOstar Omega microplate reader (BMG Labtech). Pe299R$_{CUSPER}$ and the parental Pe299R::mSc strain were grown until the mid-exponential phase (OD$_{600nm}$ ~ 0.5) in NB. Cells were collected by centrifugation (5 min at 2,000 × *g*) and washed twice with PBS. Bacterial suspensions were adjusted to an OD$_{600}$ of 0.5 and 20 µL were used to seed each well of a flat bottom 96-well microtiter plate (Costar) containing NB supplemented with increasing concentrations of IPTG (0-5 mM). Fluorescence intensity was measured in 15 min intervals in a

FLUOstar Omega microplate reader (BMG Labtech) for 20 h (Fig. S13). mClover3 fluorescence was measured using an excitation filter of 485-12 nm and an emission filter of 540-10 nm, while OD was detected with a 600 nm filter.

Growth inhibition

To determine potential interference competition between focal species and Pe299R, a double layer assay was conducted as described elsewhere [7, 8]. In brief, overnight culture of Pe299R::mSc in R2A was harvested by centrifugation (5,000 × *g* for 5 min), washed twice in PBS, and mixed in soft R2A agar (0.75% w/v agar) kept at 40°C with a temperature-controlled magnetic stirrer (MR Hei-Tec, Heidolph) to reach a concentration of OD_{600} 0.01 (top layer). This solution was quickly poured onto an agar plate containing 15 mL of R2A (bottom layer). Then, fresh cultures of each focal species grown on R2A agar were resuspended, washed twice in PBS, and adjusted to an OD_{600} of 1.0. The top layer was drop-inoculated with 2 µL of each bacterial suspension and plates were incubated at 30 °C for 2 days. As control, a 2 µL drop of 15 mg mL^{-1} gentamicin and 5 mg mL^{-1} tetracycline were deposited onto the top layer to inhibit Pe299R growth locally. The experiments were conducted twice independently with three technical replicates in each experiment.

SUPPLEMENTAL FIGURES

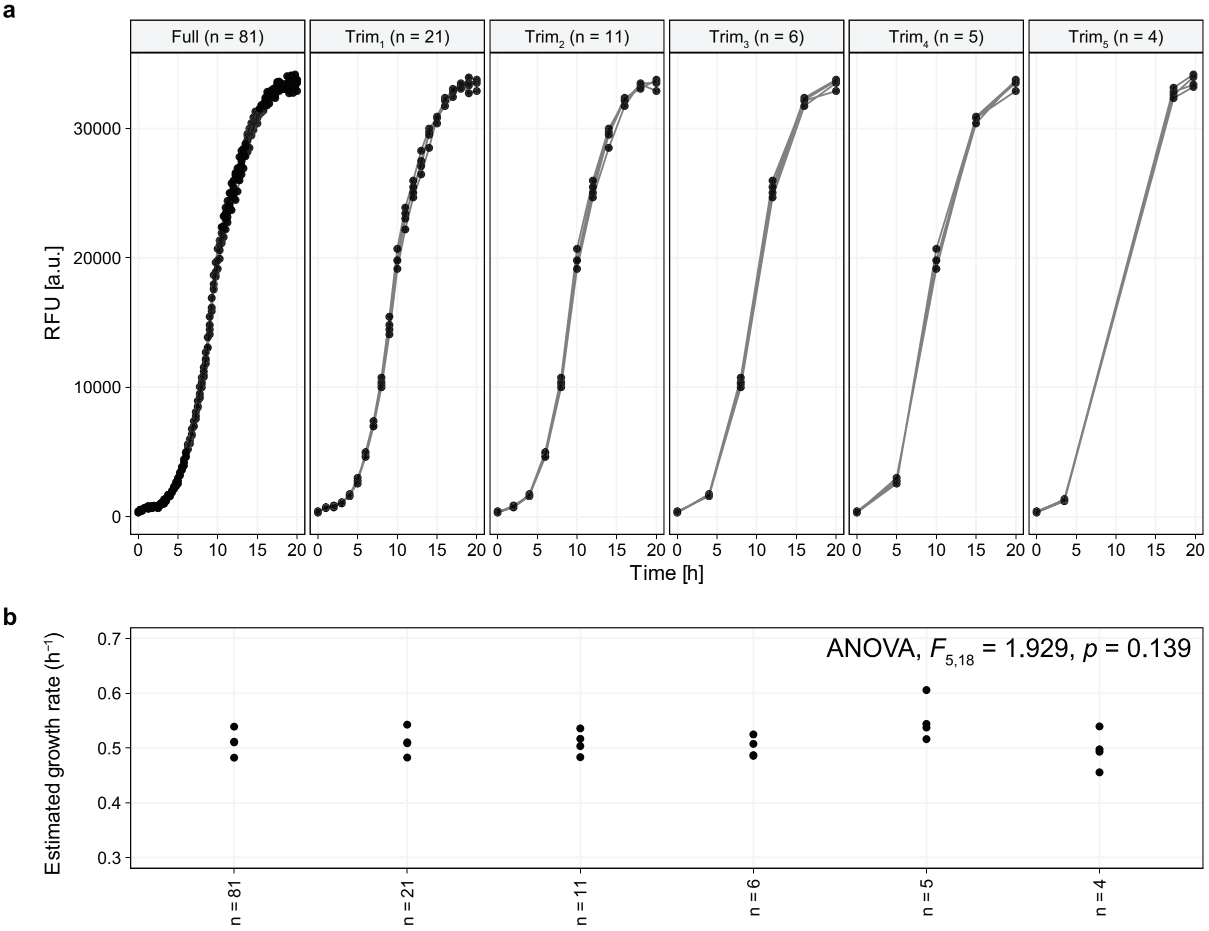

Figure S1. Temporal resolution does not influence estimated growth rates. **(a)** Growth curves of Pe299::mSc in nutrient broth with different temporal resolution after 20 h of incubation. Full: 15-min intervals (n = 81 per replicate); Trim$_1$: 1-h intervals (n = 21 per replicate); Trim$_2$: 2-h intervals (n = 11 per replicate); Trim$_3$: 4-h intervals (n = 6 per replicate); Trim$_4$: 5-h intervals (n = 5 per replicate); Trim$_5$: 0 h, 3.5 h, 17.5 h, 19.75 h (n = 4 per replicate). **(b)** Estimated growth rates (h^{-1}) from logistic functions fitted in (a) with *growthcurver*. Differences between groups were analysed by ANOVA.

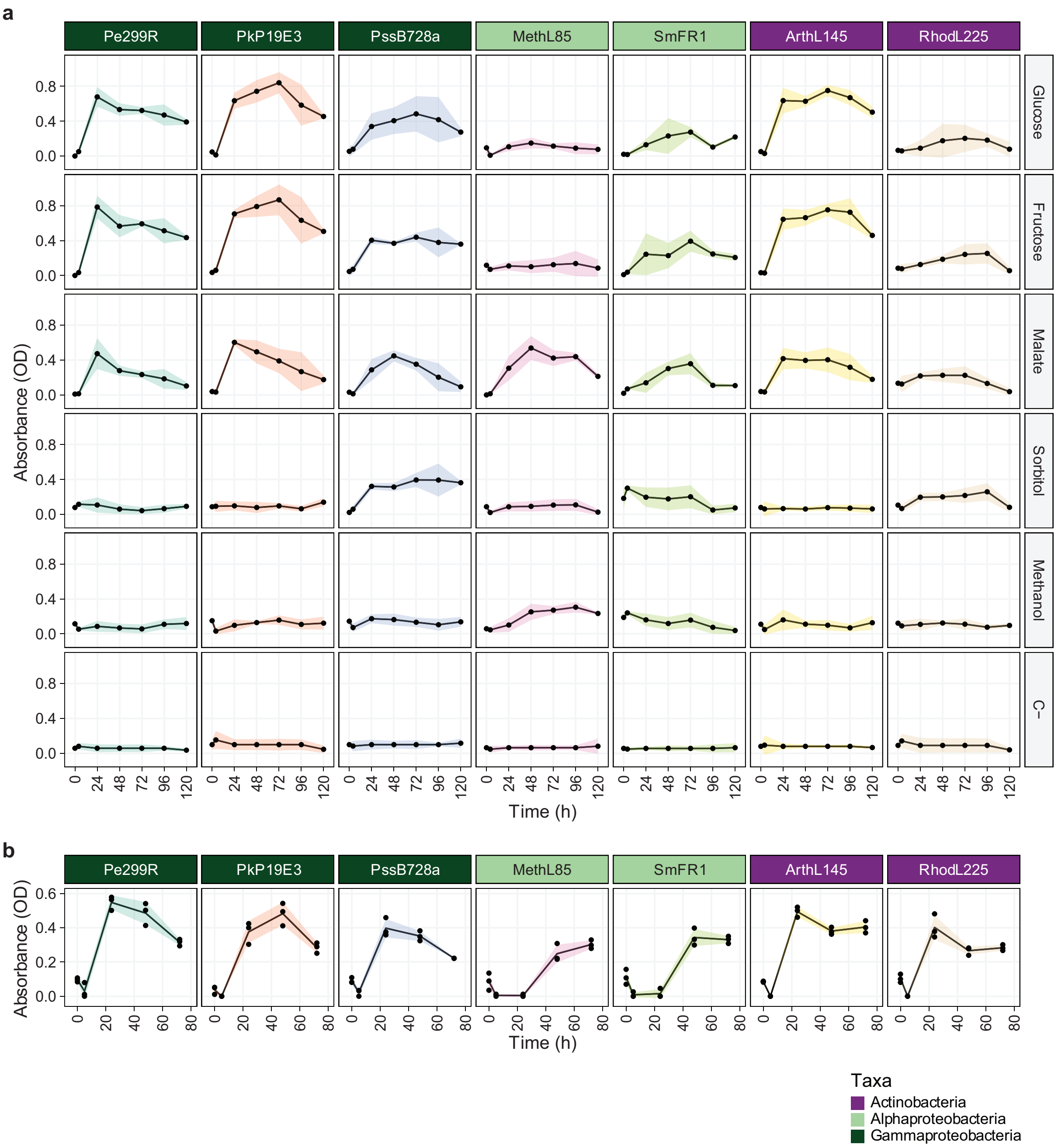

Figure S2. Growth of epiphytic bacteria in different carbon sources**. (a)** Growth of epiphytic bacteria in minimal media supplemented with 0.2% w/v of glucose, fructose, malate, sorbitol, 0.2% v/v methanol. Normalisation was made in relation to the negative control (C-). **(b)** Growth of epiphytic bacteria in minimal media supplemented with 0.125% w/v of mixed carbon sources (MM_{M5C}). Growth was measured as the absorbance at 600 nm (OD_{600}) over time. As negative control, MM without carbon sources was used (C-). Coloured area indicates the area covered by the mean ± SD at each time point.

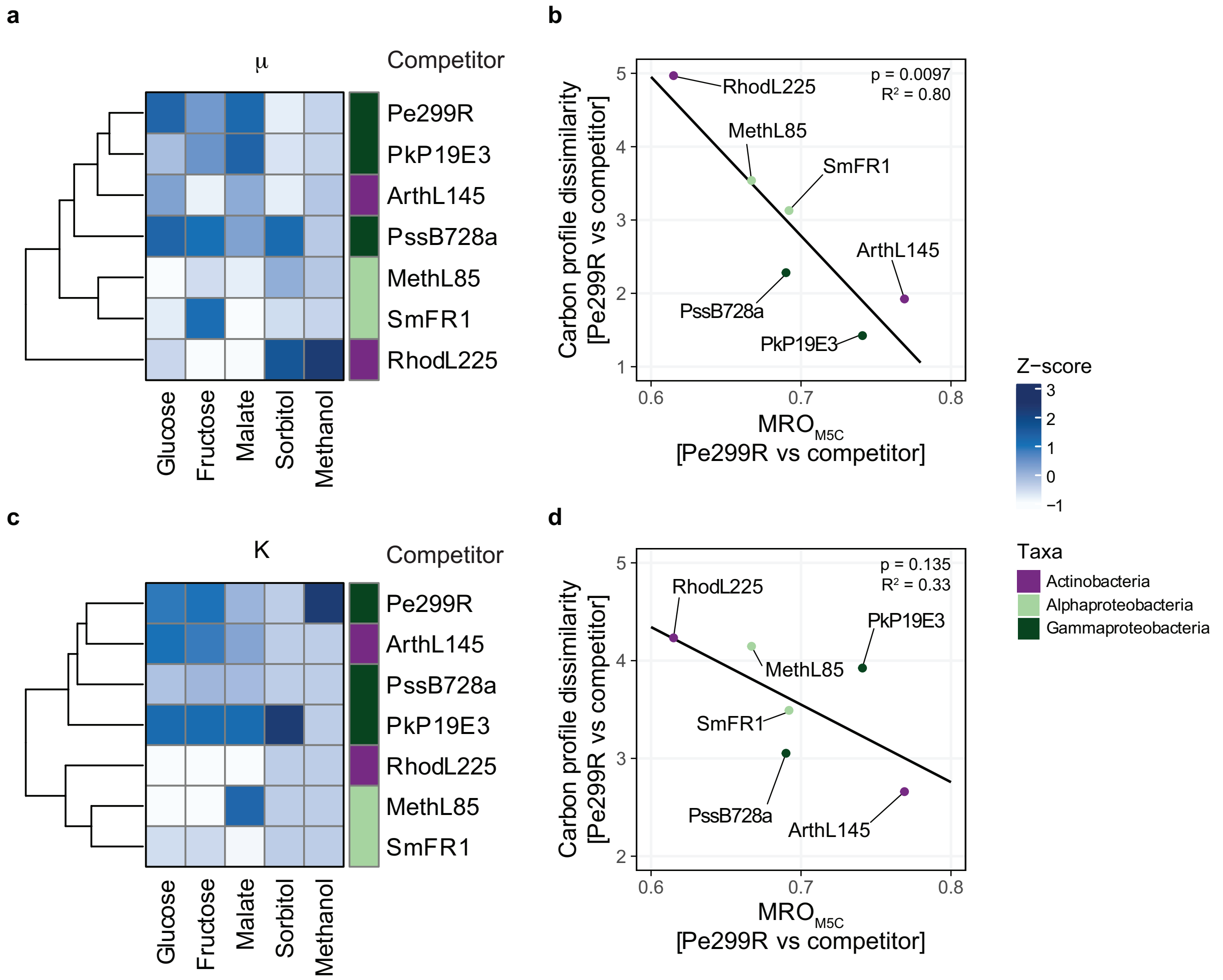

Figure S3. Carbon utilisation matrix of each bacterial strains based on **(a)** estimated growth rates (μ) or **(c)** carrying capacity (K) from growth curves in minimal medium supplemented with individual carbon sources. Values were rescaled into z-scores for complete-linkage clustering. Linear relationship of MRO$_{M5C}$ and carbon profile dissimilarity between Pe299R and a second strain based on differences in **(b)** estimated growth rates (μ) or **(d)** carrying capacity (K).

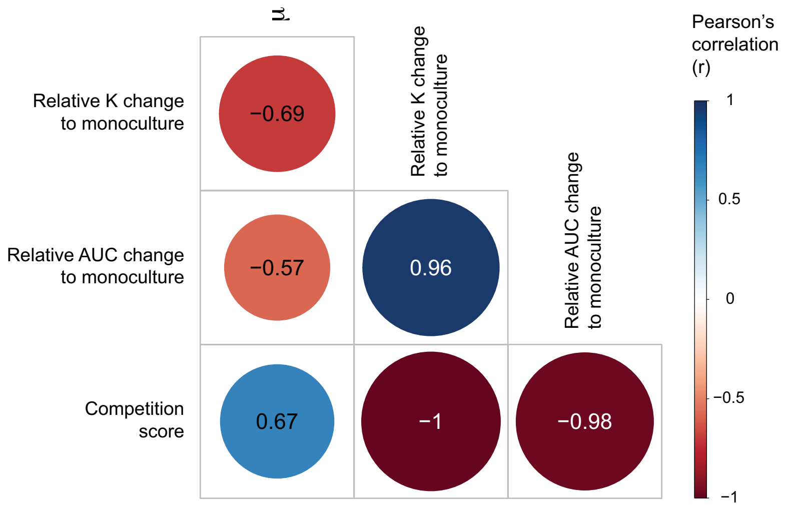

$$\text{Relative AUC change to monoculture} = \frac{AUC_{inter} - AUC_{mono}}{AUC_{mono}}$$

$$\text{Relative K change to monoculture} = \frac{K_{inter} - K_{mono}}{K_{mono}}$$

Figure S4. Comparison of competitive ability score of epiphytes and Pe299R fluorescence curve parameters. Pearson's correlation coefficients (*r*) between competition score (Eq. 1) of an epiphyte against Pe299R in $MM_{5\times C}$ and the estimated growth rates (μ), relative change in carrying capacity (relative K change), and relative change in area under the fluorescent curve (relative AUC change) of Pe299R in the presence of an epiphyte in relation to the monoculture. Relative change to monoculture was calculated as the fractional difference between K or AUC of Pe299R in interspecific competition in relation to Pe299R as monoculture.

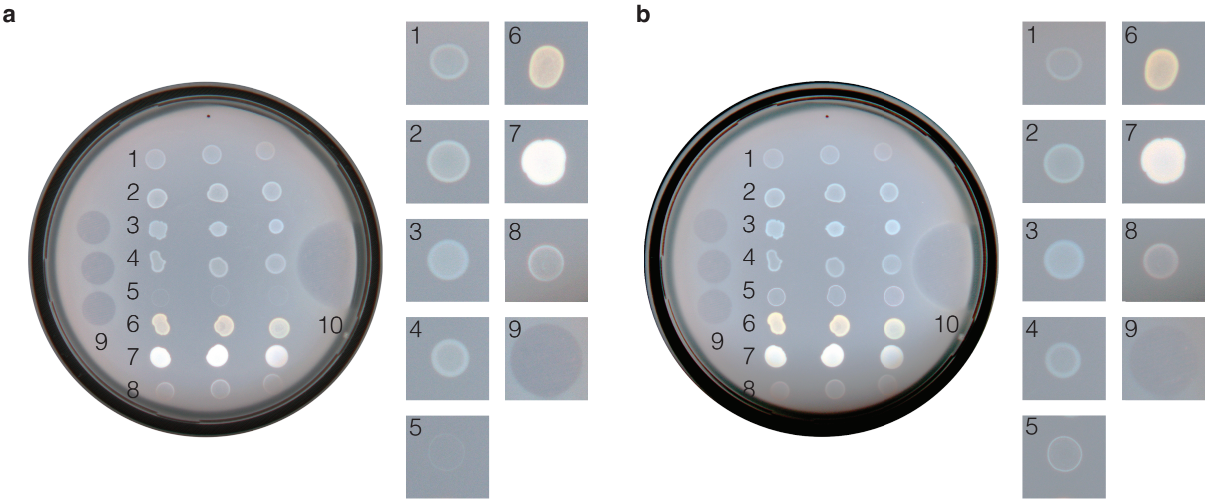

Figure S5. Growth inhibition assay. Growth of bacterial strain onto a top layer containing *Pantoea eucalypti* 299R (OD_{600} = 0.01) in R2A at **(a)** 24 and **(b)** 48 h of incubation at 30 °C. In **(a)** and **(b)**, the top layer was inoculated with: (1) Pe299R::mSc; (2) Pe299R; (3) PkP19E3; (4) PssB728a; (5) MethL85; (6) SmFR1; (7) ArthL145; (8) RhodL225; (9) Gentamicin; (10) Tetracycline.

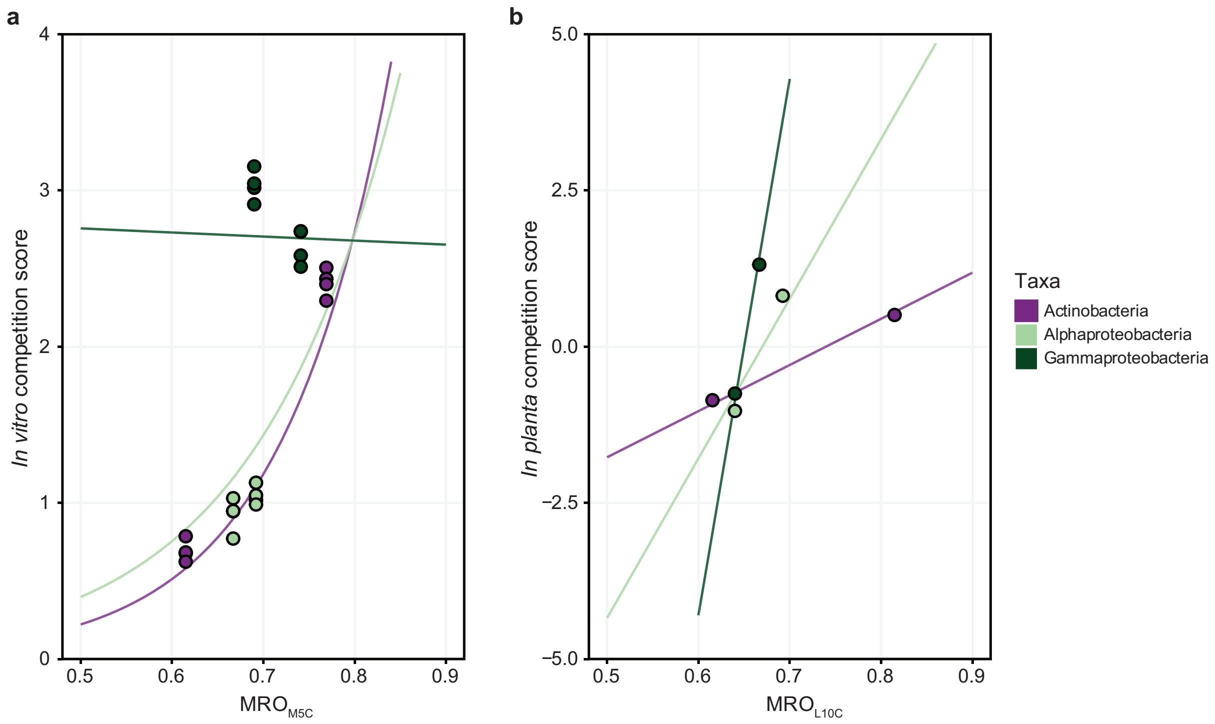

Figure S6. Resource overlap and phylogenetic distance influence competition scores of epiphytes against Pe299R**.** Regression models of competition scores explained by MRO and the phylogenetic group of an epiphyte in relation to Pe299R in **(a)** minimal medium (M5C) and in **(b)** the phyllosphere (L10C). Details of the regression models are found in Table S4 and Table S6.

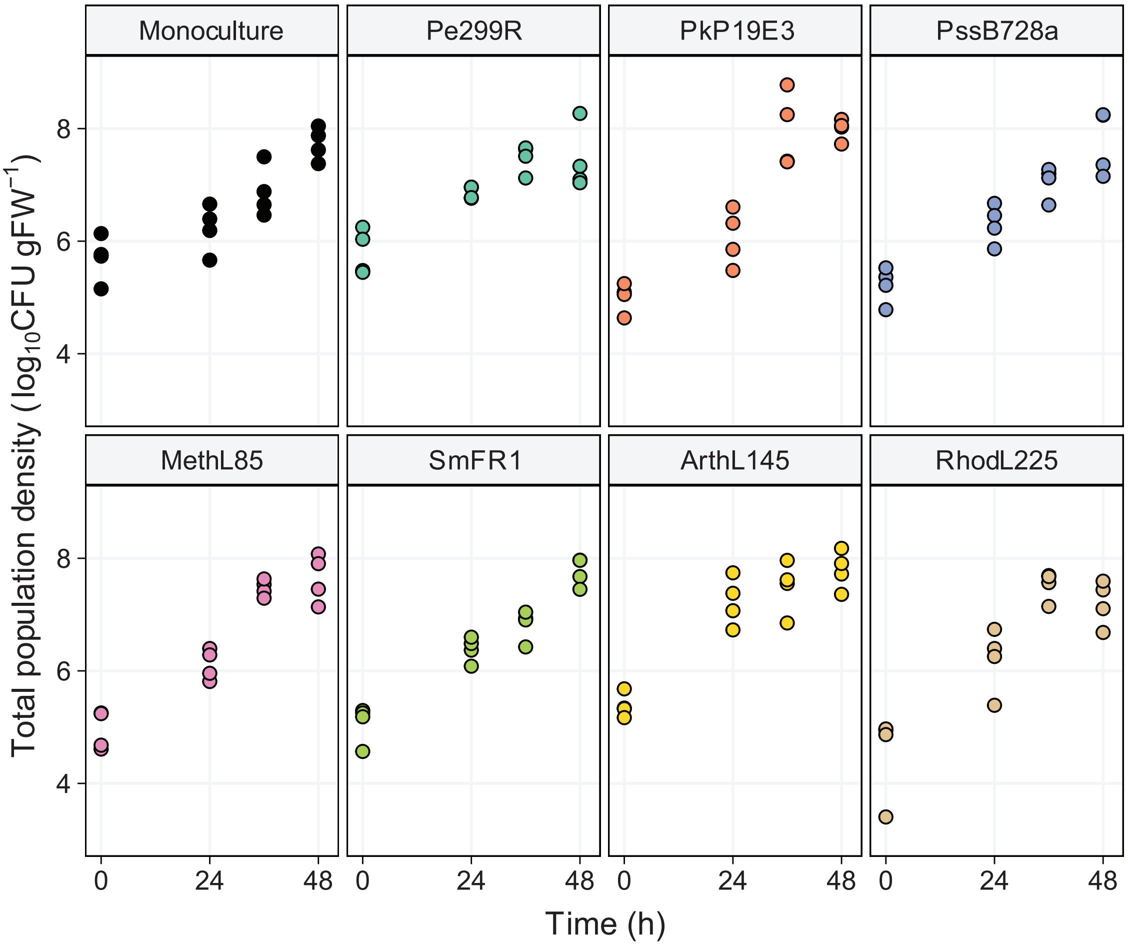

Figure S7. Total bacterial density in the phyllosphere. Each data point represents the total CFU counts per gram of fresh leaf weight (CFU gFW^{-1}) of independent plants (n = 4) at different sampling points (0, 24, 36, and 48 h). In each plot, Pe299R was co-inoculated with a second epiphyte (top label).

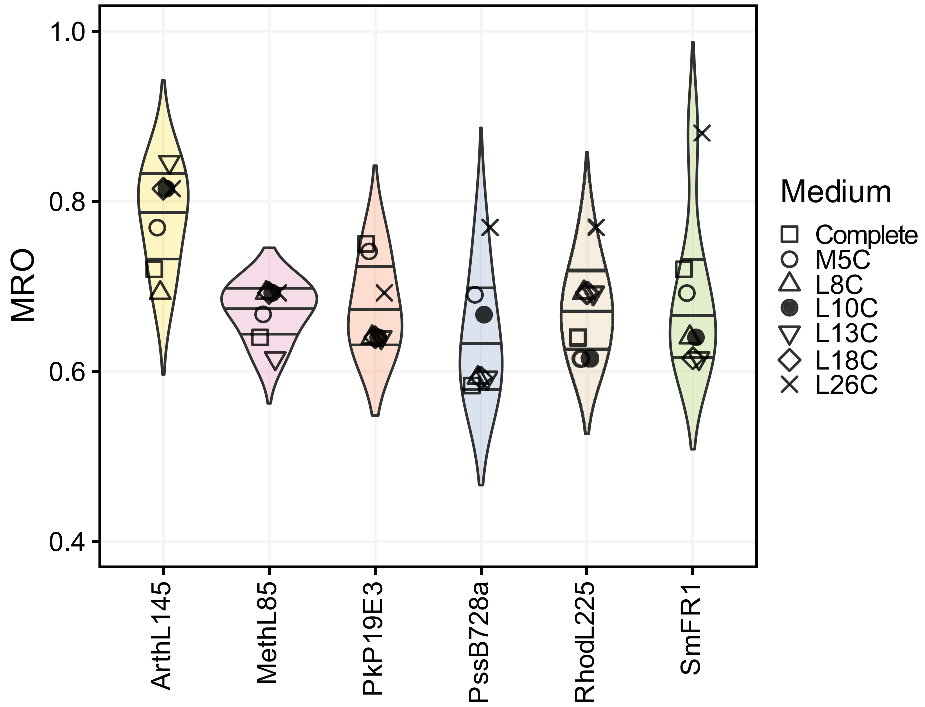

Figure S8. MRO between Pe299R and a second epiphyte in different growth compositions. MRO values from 2-spp. communities including Pe299R and a second epiphyte calculated from different environments. L10C indicated as a filled point is the medium whose MRO showed the most predictive power. A detailed list of each *in silico* medium composition can be found in Table S5.

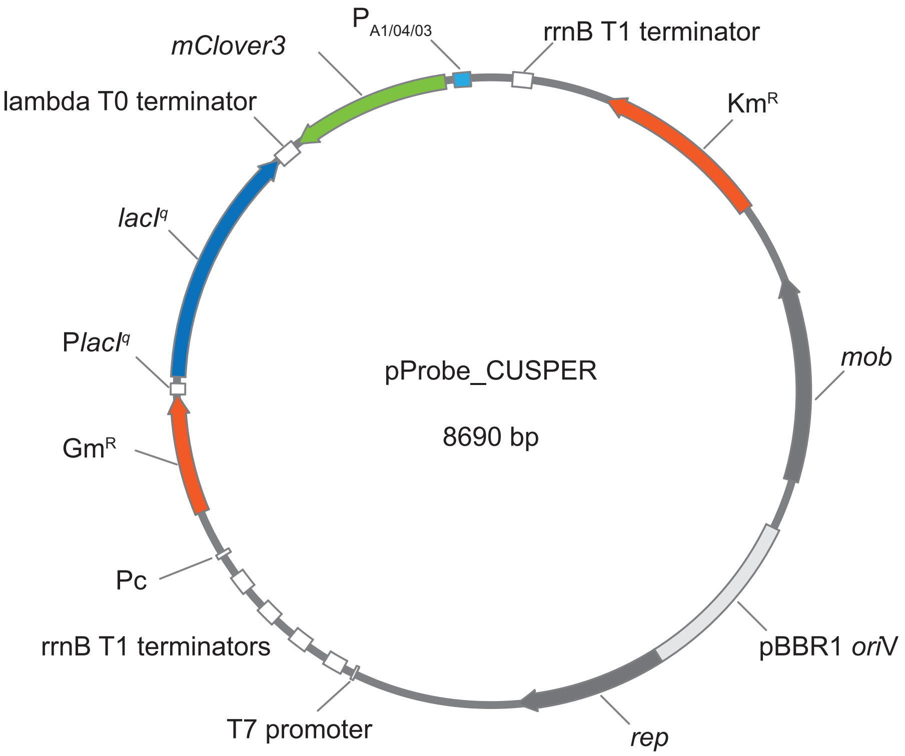

Figure S9. pProbe_CUSPER plasmid map. Plasmid-borne CUSPER bioreporter (8,690 bp). Green fluorescent protein gene variant, *mClover3*, is under the control of the *lac* promoter $P_{A1/04/03}$. Selection markers are a kanamycin resistance (Km^R) and a gentamicin resistance (Gm^R) gene. Origin of replication pBBR1 *oriV*. Genetic elements include repressor protein gene *lac*^{Iq}, replication protein gene, *rep*, and mobilisation protein genes, *Mob*. Other genetic elements such as terminators and promoters are indicated in white.

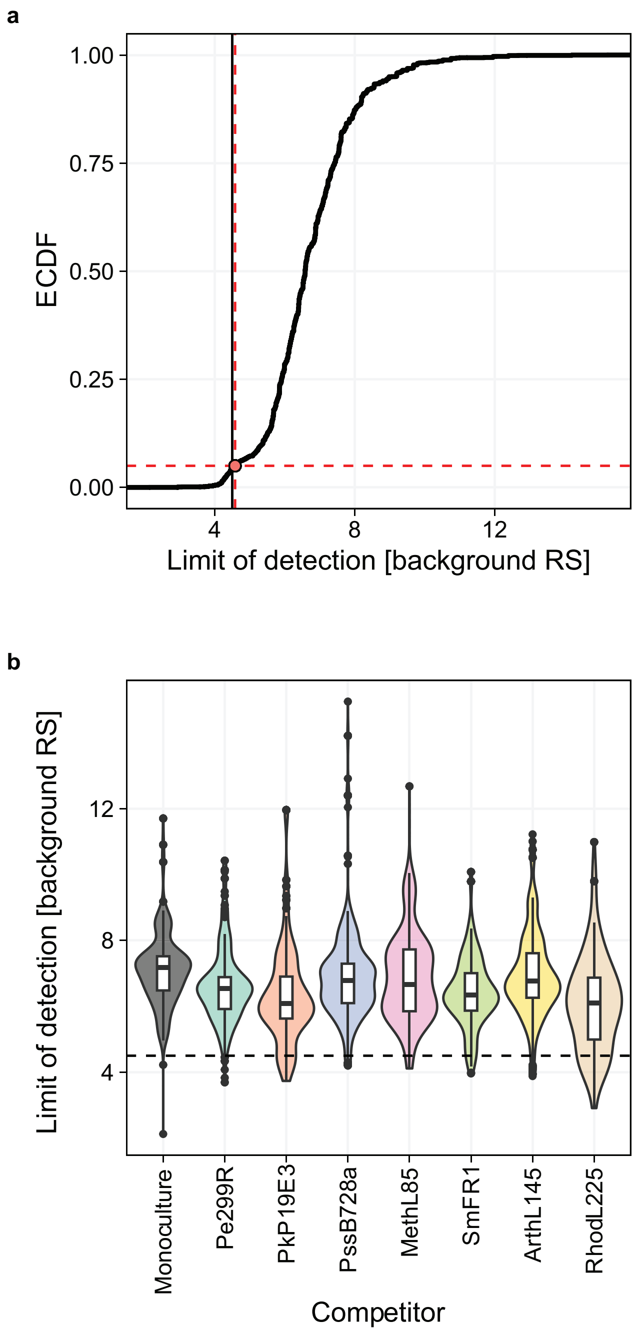

Figure S10. Limit of detection of Pe299R$_{CUSPER}$. **(a)** Empirical cumulative distribution function (ECDF) of theoretical reproductive success calculated from background fluorescence (background RS). Red dashed lines indicate the 5% threshold of background fluorescent RS (RS_{LOD} = 4.58). Continuous vertical line indicates the limit of detection set at $RS_{>4}$ = 4.50. **(b)** Distribution of background RS of each field of view per treatment group. Dashed line represents the limit of detection.

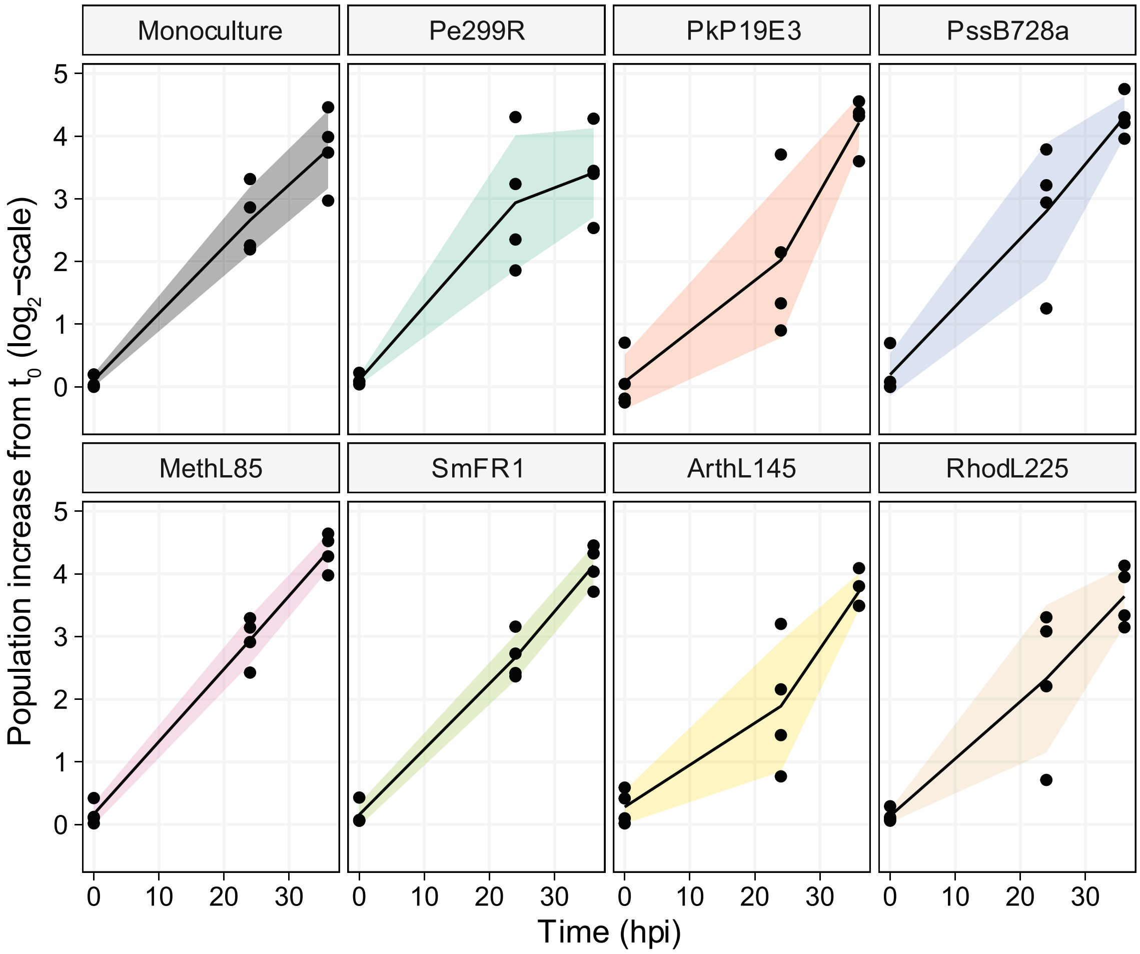

Figure S11. Increase of *Pe*299R$_{CUSPER}$ population over time in the presence of a competitor in the phyllosphere. Increase in population size of Pe299R$_{CUSPER}$ from single-cell fluorescent measurements at 0, 24, and 36 h in relation to the founder Pe299R population (population at time zero, t_0). Arabidopsis plants were co-inoculated with Pe299R$_{CUSPER}$ and a second epiphyte (top label). Each point represents the population increase in samples taken from independent plants.

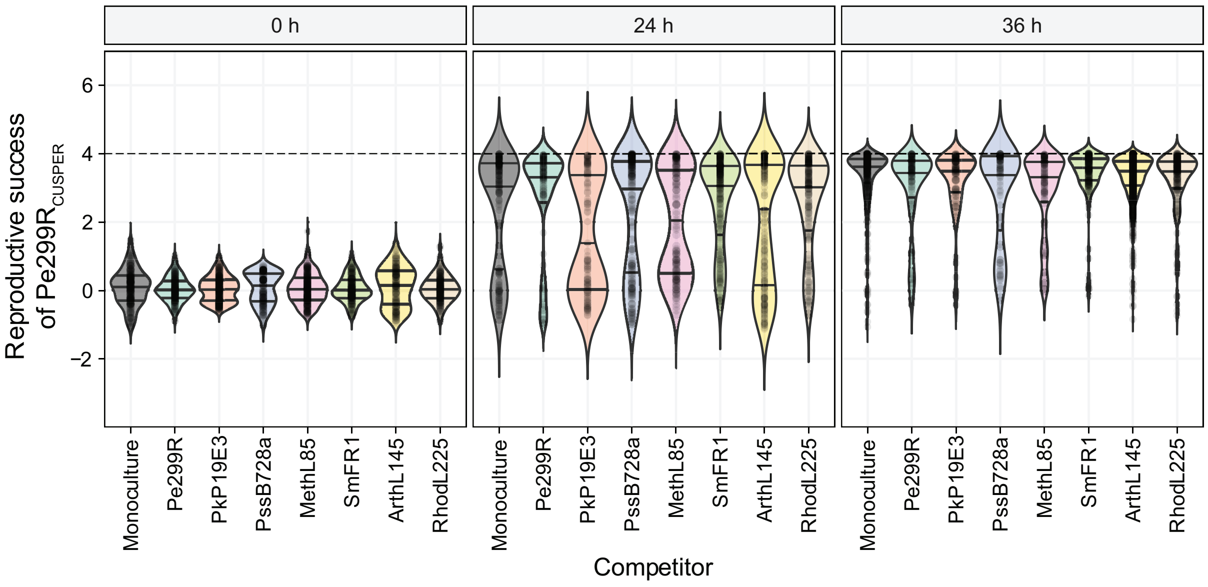

Figure S12. Single-cell reproductive success of Pe299R$_{CUSPER}$ populations in competition in the phyllosphere. Distribution of the reproductive success of single Pe299R$_{CUSPER}$ cells in the presence of an epiphyte or as monoculture at 0, 24, and 36 h post-inoculation onto arabidopsis leaves. Each violin plot indicates the median and the interquartile range.

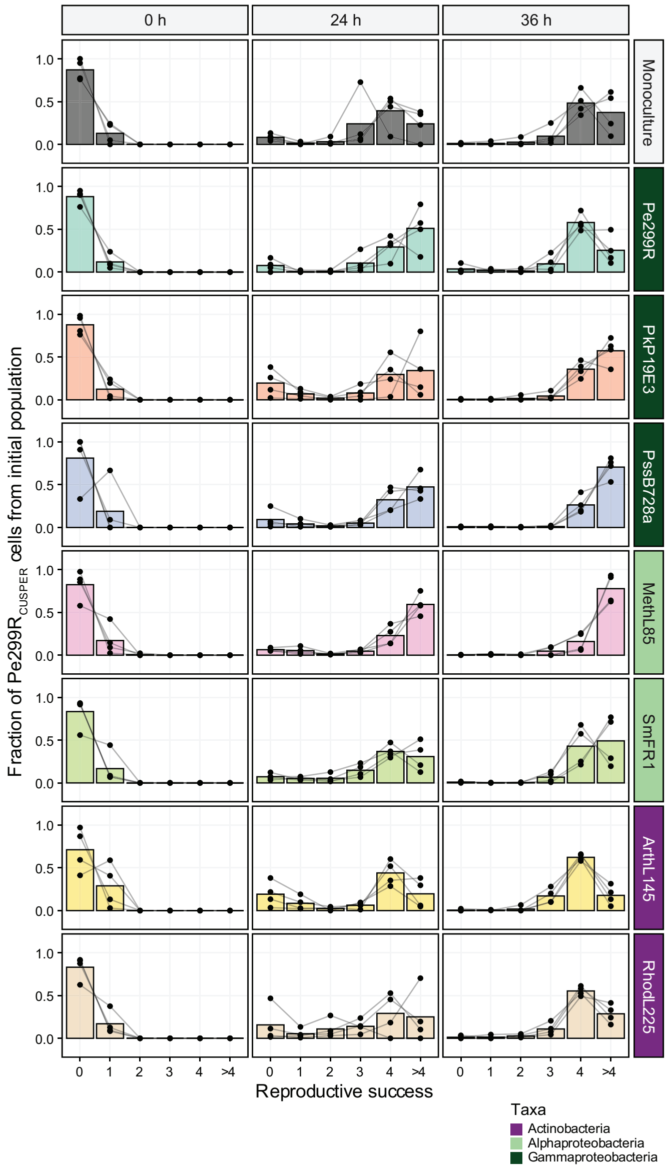

Figure S13. Changes in the composition of the Pe299R$_{CUSPER}$ populations over time in the presence of a second epiphyte. Relative fraction of the reproductive success of the founder population to the observed Pe299R$_{CUSPER}$ population at each sampling time point (0, 24, and 36 hpi) as monoculture or in the presence of a second epiphyte. The relative fractions of each biological replicate are shown and connected with a grey line. The bar represents the mean relative fraction for all replicates.

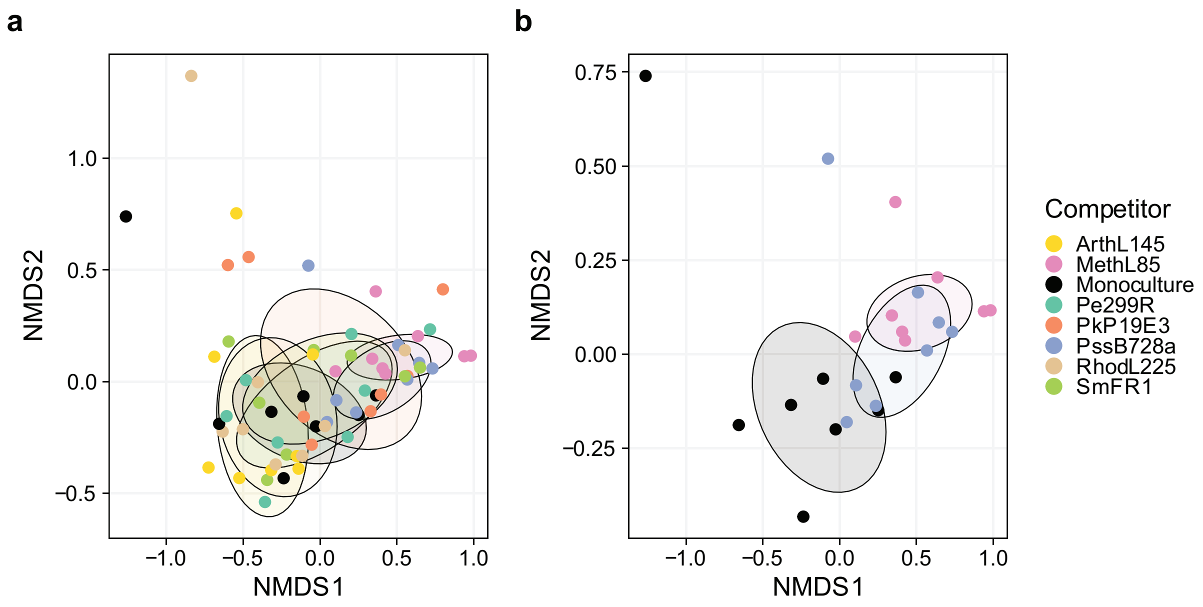

Figure S14. Non-metric multidimensional analysis of single-cell Pe299R$_{CUSPER}$ population composition in the presence of a second epiphyte. **(a)** NMDS was used to discriminate differences in population composition of Pe299R$_{CUSPER}$ at different sampling points and second epiphytes. **(b)** NMDS plot including only significantly different population compositions in relation to the monoculture.

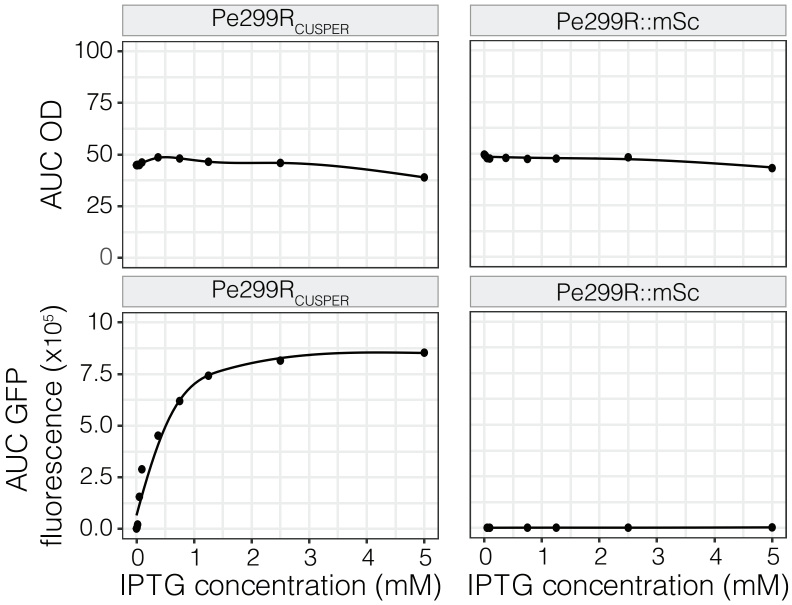

Figure S15. IPTG concentration curve for Pe299R$_{CUSPER}$ induction. Optimal concentration of IPTG for the induction of mClover3 in Pe299R$_{CUSPER}$ was evaluated in NB medium supplemented in increasing concentrations of IPTG. Optical density at 600 nm (OD) and green fluorescence were measured over time and the area under the curve (AUC) was used to estimate the biomass and fluorescence yield, respectively, of each growth condition. The parental strain Pe299R::mSc was used as a control.

SUPPLEMENTAL TABLES

Table S1. *In silico* media composition for MRO calculations. Inorganic compounds present in every medium composition are given below.

Medium	Description	Compound ID (BiGG database)	Compound name
M5C	Minimal medium 5×C	glc__D	D-Glucose
		fru	D-Fructose
		mal__L	L-Malate
		sbt__D	D-Sorbitol
		meoh	Methanol
L8C	Leaf-medium 8×C	fum	Fumarate
		mal__L	L-Malate
		glc__D	D-Glucose
		fru	D-Fructose
		glu__L	L-Glutamate
		asp__L	L-Aspartate
		sucr	Sucrose
		meoh	Methanol
L10C	Leaf-medium 10×C	fum	Fumarate
		sucr	Sucrose
		asp__L	L-Aspartate
		mal__L	L-Malate
		cit	Citrate
		glu__L	L-Glutamate
		ala__L	L-Alanine
		fru	D-Fructose
		thr__L	L-Threonine
		meoh	Methanol
L13C	Leaf-medium 13×C	fum	Fumarate
		lac__L	L-Lactate
		mal__L	L-Malate

		ala__L	L-Alanine
		fru	D-Fructose
		glc__D	D-Glucose
		glu__L	L-Glutamate
		asp__L	L-Aspartate
		chol	Choline
		4abut	4-Aminobutanoate (GABA)
		phe__L	L-Phenylalanine
		tyr__L	L-Tyrosine
		meoh	Methanol
L18C	Leaf-medium 18×C	fum	Fumarate
		mal__L	L-Malate
		glc__D	D-Glucose
		fru	D-Fructose
		glu__L	L-Glutamate
		ala__L	L-Alanine
		asp__L	L-Aspartate
		sucr	Sucrose
		gal	D-Galactose
		arab__L	L-Arabinose
		mnl	D-Mannitol
		cit	Citrate
		thr__L	L-Threonine
		gln__L	L-Glutamine
		lys__L	L-Lysine
		asn__L	L-Asparagine
		4abut	4-Aminobutanoate (GABA)
		meoh	Methanol
L26C	Leaf-medium 26×C	ala__L	L-Alanine
		asp__L	L-Aspartate
		asn__L	L-Asparagine
		chol	Choline
		citr__L	L-Citrulline

		fru	D-Fructose
		fum	Fumarate
		4abut	4-Aminobutanoate (GABA)
		glc__D	D-Glucose
		glu__L	L-Glutamate
		gln__L	L-Glutamine
		gly	Glycine
		ile__L	L-Isoleucine
		lac__L	L-Lactate
		leu__L	L-Leucine
		mal__L	L-Malate
		meoh	Methanol
		inost	Myo-Inositol
		orn	Ornithine
		phe__L	L-Phenylalanine
		succ	Succinate
		sucr	Sucrose
		thr__L	L-Threonine
		trp__L	L-Tryptophan
		tyr__L	L-Tyrosine
		val__L	L-Valine

Inorganic compounds: Ca^{2+}, Cl^{-}, Co^{2+}, Cu^{2+}, Fe^{2+}, Fe^{3+}, H_2O, H^{+}, K^{+}, Mg^{2+}, Mn^{2+}, Molybdate, Na^{+}, Ammonium, Ni^{2+}, O_2, Phosphate, Sulfate, Zn^{2+}.

Table S2. Growth parameters of each epiphyte growing in minimal medium supplemented with one (MM + C) or a mix of carbon sources ($MM_{5\times C}$).

**Strain**	**Growth medium**	**K (OD)**	**µ (h^{-1})**
ArthL145	MM + Fructose	0.560	0.614
	MM + Glucose	0.560	1.066
	MM + Malate	0.297	0.702
	MM + Methanol	0.083	0.157
	MM + Sorbitol	0.000	0.000
	MM_{M5C}	0.426	0.953
MethL85	MM + Fructose	0.030	0.792
	MM + Glucose	0.105	0.097
	MM + Malate	0.403	0.263
	MM + Methanol	0.225	0.146
	MM + Sorbitol	0.066	0.120
	MM_{M5C}	0.298	0.366
Pe299R	MM + Fructose	0.595	1.400
	MM + Glucose	0.534	1.684
	MM + Malate	0.275	1.212
	MM + Methanol	0.000	0.008
	MM + Sorbitol	0.000	0.000
	MM_{M5C}	0.419	0.937
PkP19E3	MM + Fructose	0.633	1.447
	MM + Glucose	0.597	0.826
	MM + Malate	0.392	1.274
	MM + Methanol	0.099	0.023
	MM + Sorbitol	0.000	0.019
	MM_{M5C}	0.383	0.417
PssB728a	MM + Fructose	0.332	1.826
	MM + Glucose	0.286	1.706

	MM + Malate	0.264	0.760
	MM + Methanol	0.086	0.116
	MM + Sorbitol	0.315	0.261
	MM_{M5C}	0.301	0.959
RhodL225	MM + Fructose	0.068	0.166
	MM + Glucose	0.068	0.620
	MM + Malate	0.121	0.177
	MM + Methanol	0.017	2.363
	MM + Sorbitol	0.092	0.322
	MM_{M5C}	0.317	0.709
SmFR1	MM + Fructose	0.216	1.858
	MM + Glucose	0.207	0.445
	MM + Malate	0.182	0.142
	MM + Methanol	0.000	0.000
	MM + Sorbitol	0.110	0.032
	MM_{M5C}	0.328	0.751

192

Table S3. Growth parameters of Pe299R::mSc from red fluorescence curves in the presence of a competitor or as monoculture.

**Competitor**	**K (RFU)**	**µ (h^{-1})**	**AUC (RFU)**	**Competition score**
Monoculture	1363.69	0.26	12859.26	0.194
ArthL145	519.99	0.54	7586.59	2.409
MethL85	1041.74	0.32	11797.01	0.924
Pe299R	663.26	0.37	8105.26	1.947
PkP19E3	454.27	0.47	6172.73	2.643
PssB728a	354.60	0.36	5129.68	3.032
RhodL225	1139.11	0.34	13402.83	0.692
SmFR1	991.81	0.39	12181.51	1.046

Table S4. Summary of regression analysis of competitive scores *in vitro*.

**Model (linear regression)**	***df***	**RSE**	R^2	η^2	***F***	***p***
Competition score ~ MRO_{M5C}	22	0.70	0.46	0.48	20.7	0.0002
**Predictor**	**df**	**SE**	**β**	**95% CI**	***t***	***p***
Intercept	22	2.00	-7.28	[-11.43, -3.13]	-3.64	< 0.05
MRO_{M5C}	22	2.87	13.04	[7.09, 18.99]	4.55	< 0.05

**Model (linear regression)**	***df***	**RSE**	R^2	η^2	***F***	***p***
Competition score ~ MRO (without PssB728a)	18	0.36	0.81	0.823	83.45	< 0.05
**Predictor**	**df**	**SE**	**β**	**95% CI**	***t***	***p***
Intercept	18	1.04	-7.97	[-10.16, -5.77]	-7.63	< 0.05
MRO_{M5C}	18	1.49	13.65	[10.51, 16.78]	9.14	< 0.05

**Model (GLM, Gamma distribution, log link)**	***df***	**SE**	**Null Deviance**	**Residual Deviance**	**Pseudo-**R^2	***F***	***p***
Competition score ~ MRO × PD	20	0.042	7.60	0.80	0.89	9.61	0.0056
**Predictor**	**df**	**SE**	**β**	**95% CI**	***t***	***p***	
Intercept	20	4.10	8.88	[0.98, 16.74]	2.17	0.043	
MRO_{M5C}	20	5.73	-9.90	[-20.89, 1.18]	-1.73	0.099	
PD	20	5.64	-19.19	[-29.93, -8.40]	-3.41	0.0028	
MRO_{M5C} × PD	20	7.87	24.06	[8.92, 39.08]	3.06	0.0062	

Table S5. Summary of regression analysis of competitive scores *in planta*.

**Model (y ~ x)**	***df***	**RSE**	R^2	***F***	***p***
Competition score ~ PD	4	1.09	-0.19	0.20	0.6763
Competition score ~ PD × $MRO_{Complete}$	2	1.05	-0.11	0.84	0.5835
Competition score ~ PD × MRO_{M5C}	2	0.91	0.17	1.33	0.4556
Competition score ~ PD × MRO_{L8C}	2	1.15	-0.32	0.60	0.6747
**Competition score ~ PD ×** $\mathbf{MRO_{L10C}}$	**2**	**0.28**	**0.92**	**20.15**	**0.0477**
Competition score ~ PD × MRO_{L13C}	2	1.05	-0.10	0.85	0.5809
Competition score ~ PD × MRO_{L18C}	2	1.20	-0.43	0.50	0.7202
Competition score ~ PD × MRO_{L26C}	2	1.13	-0.27	0.65	0.6543

**Predictor (Competition score ~ PD ×** $\mathbf{MRO_{L10C}}$**)**	***df***	**SE**	β	**95% CI**	η^2	***t***	***p***
Intercept	2	19.14	-114.05	[-196.42, -31.68]		-5.96	0.027
MRO_{L10C}	2	29.29	176.62	[50.61, 302.63]	0.390	6.03	0.026
PD	2	25.44	142.94	[33.49, 252.39]	0.170	5.62	0.030
MRO_{L10C} × PD	2	38.90	-222.77	[-390.15, -55.39]	0.530	-5.73	0.029

Table S6. Summary of PERMANOVA on Pe299R$_{\text{CUSPER}}$ populations.

Model	Term	*df*	SS	R^2	*F*	*p*
Full Model	Time	1	0.585	0.09967	8.695	0.001
	Competitor	7	1.583	0.2698	3.363	0.002
	Residual	55	3.699	0.6305	n.a.	n.a.
	Total	63	5.868	1.0000	n.a.	n.a.
Monoculture vs PssB728a	Competitor	1	0.324	0.243	4.498	0.011
	Residual	14	1.008	0.757	n.a.	n.a.
	Total	15	1.331	1.000	n.a.	n.a.
Monoculture vs MethL85	Competitor	1	0.532	0.358	7.802	0.003
	Residual	14	0.954	0.642	n.a.	n.a.
	Total	15	1.486	1.000	n.a.	n.a.
24 h vs 36 h	Time	1	0.585	0.100	6.864	0.003
	Residual	62	5.283	0.900	n.a.	n.a.
	Total	63	5.868	1.000	n.a.	n.a.

Table S7. Primers used to construct pProbe_CUSPER.

**Name**	**Target**	**Sequence (5'-3')[a]**	***tm* (°C)[b]**
Plac_fw	$P_{A1/04/03}$ promoter	tcctcgcccttgctcatAAATTGTTATCCGCTCACAATTG	55
Plac_rv	$P_{A1/04/03}$ promoter	tgccactcatcgcagtactGAAAATTTATCAAAAAGAGTG	47
fp_fw	*mClover3* gene	tgagcggataacaatttATGAGCAAGGGCGAGGAGCTG	64
fp_rv	*mClover3* gene	actggaaagcgggcagtgaATTCTCACCAATAAAAAACG	50
lacIq_fw	*lacIq* repressor gene	aagtaccgccacctaaGACACCATCGAATGGTGCAAAACC	61
lacIq_rv	*lacIq* repressor gene	gtttttattggtgagaatTCACTGCCCGCTTTCCAGTCG	62
gmR_fw	*gmR* selection gene	aggaattggggatcggaagcttTTGACATAAGCCTGTTCGG	53
gmR_rv	*gmR* selection gene	ccattcgatggtgtcTTAGGTGGCGGTACTTGGGTCG	61

[a] Capital letters indicate complementary sequences to the original PCR target; lower cases indicate complementary sequences to the destination sequence (overhang).

[b] The *tm* refers to the melting temperature of the original target sequence.

REFERENCES

1. Gibson DG, Young L, Chuang R-Y, Craig Venter J, Hutchison CA, Smith HO. Enzymatic assembly of DNA molecules up to several hundred kilobases. Nature Methods. 2009; 6: 343–345
2. Schlechter RO, Jun H, Bernach M, Oso S, Boyd E, Muñoz-Lintz DA, et al. Chromatic Bacteria - A Broad Host-Range Plasmid and Chromosomal Insertion Toolbox for Fluorescent Protein Expression in Bacteria. Front. Microbiol. 2018; 9: 3052.
3. Klausen M, Heydorn A, Ragas P, Lambertsen L, Aaes-Jørgensen A, Molin S, et al. Biofilm formation by *Pseudomonas aeruginosa* wild type, flagella and type IV pili mutants. Mol. Microbiol. 2003; 48: 1511–1524.
4. Tecon R, Leveau JHJ. The mechanics of bacterial cluster formation on plant leaf surfaces as revealed by bioreporter technology. Environ. Microbiol. 2012; 14: 1325–1332.
5. Remus-Emsermann MNP, Leveau JHJ. Linking environmental heterogeneity and reproductive success at single-cell resolution. ISME J. 2010; 4: 215–222.
6. Gonzales MF, Brooks T, Pukatzki SU, Provenzano D. Rapid protocol for preparation of electrocompetent *Escherichia coli* and *Vibrio cholerae*. J. Vis. Exp. 2013; 80:50684.
7. Zengerer V, Schmid M, Bieri M, Müller DC, Remus-Emsermann MNP, Ahrens CH, et al. F9: A Potent Antagonist against Phytopathogens with Phytotoxic Effect in the Apple Flower. Front. Microbiol. 2018; 9: 145.
8. Helfrich EJN, Vogel CM, Ueoka R, Schäfer M, Ryffel F, Müller DB, et al. Bipartite interactions, antibiotic production and biosynthetic potential of the Arabidopsis leaf microbiome. Nat. Microbiol. 2018; 3: 909–919.
